# Mix and measure II: joint high-energy laboratory powder diffraction and microtomography for cement hydration studies

**DOI:** 10.1107/S1600576724004527

**Published:** 2024-07-04

**Authors:** Jaime Fernandez-Sanchez, Ana Cuesta, Shiva Shirani, Cinthya Redondo-Soto, Angeles G. De la Torre, Isabel Santacruz, Ines R. Salcedo, Laura Leon-Reina, Miguel A. G. Aranda

**Affiliations:** a Departamento de Química Inorgánica, Cristalografía y Mineralogía Universidad de Málaga 29071Málaga Spain; b Servicios Centrales de Apoyo a la Investigación Universidad de Málaga 29071Málaga Spain; HPSTAR and Harbin Institute of Technology, People’s Republic of China

**Keywords:** Portland cements, cement blends, *in situ* studies, Rietveld quantitative phase analysis, X-ray imaging, filler effect

## Abstract

Portland cements (PCs) and cement blends are multiphase materials of different fineness, and quantitatively analysing their hydration pathways is very challenging. The dissolution (hydration) of the initial crystalline and amorphous phases must be determined, as well as the formation of labile (such as ettringite), reactive (such as portlandite) and amorphous (such as calcium silicate hydrate gel) components. The microstructural changes with hydration time must also be mapped out. To address this robustly and accurately, an innovative approach is being developed based on *in situ* measurements of pastes without any sample conditioning. Data are sequentially acquired by Mo *K*α_1_ laboratory X-ray powder diffraction (LXRPD) and microtomography (µCT), where the same volume is scanned with time to reduce variability. Wide capillaries (2 mm in diameter) are key to avoid artefacts, *e.g.* self-desiccation, and to have excellent particle averaging. This methodology is tested in three cement paste samples: (i) a commercial PC 52.5 R, (ii) a blend of 80 wt% of this PC and 20 wt% quartz, to simulate an addition of supplementary cementitious materials, and (iii) a blend of 80 wt% PC and 20 wt% limestone, to simulate a limestone Portland cement. LXRPD data are acquired at 3 h and 1, 3, 7 and 28 days, and µCT data are collected at 12 h and 1, 3, 7 and 28 days. Later age data can also be easily acquired. In this methodology, the amounts of the crystalline phases are directly obtained from Rietveld analysis and the amorphous phase contents are obtained from mass-balance calculations. From the µCT study, and within the attained spatial resolution, three components (porosity, hydrated products and unhydrated cement particles) are determined. The analyses quantitatively demonstrate the filler effect of quartz and limestone in the hydration of alite and the calcium aluminate phases. Further hydration details are discussed.

## Abstract 2

Laboratory powder diffraction and microtomography techniques are sequentially used in the same volume of the same sample to study the process of cement hydration with time.

## 1. Introduction

Understanding the hydration of Portland cements (PCs) and PC blends (mixtures with other materials) is very challenging because they are multicomponent (Taylor, 1997 ▸; Hewlett & Liska, 2017 ▸). The low crystallinity of some phases is the main problem for X-ray powder diffraction characterization, while the small sizes of pores and of some particles, and the low contrast in the X-ray absorption of the different components, are the main issues for X-ray imaging studies. PC CEM I type is composed of Portland clinker with two minor additions: (i) a sulfate carrier (gypsum/bassanite/anhydrite) to delay the hydration of tricalcium aluminate which contributes to regulate setting, and (ii) limestone (mainly calcite) to optimize the rheology without degrading the mechanical performance. However, most of the Portland-based cements used have other additions. PC clinker replacement by supplementary cementitious materials (SCMs) (Juenger & Siddique, 2015 ▸; Juenger *et al.*, 2019 ▸; Snellings *et al.*, 2023 ▸), like slag, fly ashes, kaolinitic calcined clays or natural pozzolans, is key to decreasing the CO_2_ footprint of the resulting mortars and concretes. Naturally, these additions add complexity to the hydration of the resulting binders.

PCs and PC–SCM blends are multiphase systems with different elemental compositions, phases (type and content) and textural properties, such as specific surface areas and particle size distributions. The hydration processes happen under various conditions, including water-to-binder mass ratio (w/b), temperature and pressure. The complexity of binder hydration stems from the evolution of the phases and the resulting microstructures. After initial partial dissolution and supersaturation, a set of coupled reactions leads to the precipitation of several hydrates within evolving microstructures (Taylor, 1997 ▸; Hewlett & Liska, 2017 ▸). Hence, a large number of analytical techniques are employed to study the hydration processes with accuracy. The most common techniques, including laboratory X-ray powder diffraction (LXRPD), have been described in detail in a book that was published in 2016 (Scrivener *et al.*, 2016 ▸) and which is currently the standard in the field. However, X-ray microcomputed tomography (µCT) (Maire & Withers, 2014 ▸; Brisard *et al.*, 2020 ▸; Withers *et al.*, 2021 ▸) was not included in that book.

Cement notation will be used hereafter (Taylor, 1997 ▸). Before being mixed with water, PCs consist of more than six crystalline components: 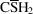 (CaSO_4_·2H_2_O, gypsum and/or other calcium sulfates), C_3_S (Ca_3_SiO_5_, alite), C_3_A (Ca_3_Al_2_O_6_, tricalcium aluminate), C_4_AF (Ca_4_Al_2_Fe_2_O_10_, tetracalcium aluminoferrite), Cc (CaCO_3_, calcite/limestone) and C_2_S (Ca_2_SiO_4_, belite), ordered by their hydration reaction rates. After water mixing and at different timescales, more than five new hydration products are formed: AFt [Ca_6_Al_2_(SO_4_)_3_(OH)_12_·26H_2_O, ettringite], C–S–H gel (nanocrystalline, nearly amorphous, calcium silicate hydrate), CH [Ca(OH)_2_, portlandite], Fe–Si–H (amorphous iron–siliceous hydro­garnet), Hc [Ca_4_Al_2_(OH)_13_(CO_3_)_0.5_(H_2_O)_5.5_, hemi­carbo­aluminate] and Mc [Ca_4_Al_2_(OH)_12_(CO_3_)(H_2_O)_5_, mono­carboaluminate], ordered by their approximate formation hydration kinetics. The Hc and Mc phases are AFm-type crystalline phases, but other AFm phases, with variable stoichiometries in the interlayer space, are also known, including amorphous variants (Balonis & Glasser, 2009 ▸; Matschei *et al.*, 2007 ▸).

On the one hand, time-dependent quantification of the crystalline components for neat PC hydration is nowadays well established (Aranda *et al.*, 2012 ▸, 2017 ▸, 2019 ▸; Scrivener *et al.*, 2016 ▸; Jansen *et al.*, 2012 ▸; Qoku *et al.*, 2023 ▸). The overall amorphous content can be determined using external (Jansen, Goetz-Neunhoeffer *et al.*, 2011 ▸) or internal (De la Torre *et al.*, 2001 ▸) standard methodologies. However, obtaining the degree of hydration of SCMs in PC–SCM blends is very challenging (Juenger & Siddique, 2015 ▸; Juenger *et al.*, 2019 ▸; Snellings *et al.*, 2023 ▸). This is due to several factors, including the presence of several amorphous components: C–S–H gel, Fe–Si–H, free water (FW), the amorphous phase within the SCM (for instance, SiO_2_ in natural pozzolans) and C–(A)–S–H gel. In addition, X-ray diffraction with a flat sample geometry is prone to some systematic errors, such as preferred orientation and water/solid bleeding. Specifically, for cements, special care has to be exercised to avoid carbonation of CH, as accurately measuring portlandite content is critical to determine the pozzolanic reaction degree, *i.e.* the reaction between siliceous/aluminium oxides with Ca(OH)_2_ to yield C–(A)–S–H gel and other hydrates, such as AFt, Hc and Mc, depending upon the SCMs employed. It is thus important to develop experimental approaches which minimize experimental errors.

On the other hand, even after 15 years of research, analysing the time evolution of components and microstructures in cement hydration by µCT (Withers *et al.*, 2021 ▸) remains a significant challenge. The uses of µCT in cements have been reviewed elsewhere (da Silva, 2018 ▸; du Plessis & Boshoff, 2019 ▸; Brisard *et al.*, 2020 ▸; Kong *et al.*, 2020 ▸; Sugiyama & Promentilla, 2021 ▸; Chung *et al.*, 2019 ▸). Here, it is noted that synchrotron µCT, in the different modalities (absorption-based, phase-contrast, ptychography *etc.*) (Aranda, 2016 ▸; Shirani *et al.*, 2023 ▸; Qoku *et al.*, 2023 ▸), yields more information than laboratory µCT, but access to this set of techniques is very limited and demanding. The spatial resolution of synchrotron µCT, for hydration studies, depends upon the chosen modality and tested field of view. The voxel sizes can be two to three times smaller. The spatial resolution of laboratory µCT for hydration studies also depends upon the measured field of view and it is usually 2–3 µm or worse. Features smaller than the spatial resolution cannot be determined in conventional laboratory-based X-ray imaging, which is a problem because (i) the gel porosity of cement pastes ranges from 5 to 100 nm, (ii) the capillary porosity varies between 100 nm and a few micrometres, and (iii) several cement components have quite a significant fraction of particle sizes smaller than 2 µm. Nevertheless, research on cement hydration using laboratory µCT is increasing as this technique does not require sample preparation which is known to alter the labile microstructures of hydrating binders.

The research presented here is aimed at the *in situ* analysis of cement hydration by combining LXRPD and µCT. These techniques require no sample preparation and allow automation of data analysis. Our approach is based on sequential analysis of the same volume, which imposes constraints on the results. This approach helps to deal with the complexity of the studied problem. The reported investigation is part of a long-term project finally to address the development of an accurate methodology to analyse PC–SCM blends, including the analysis and understanding of the pozzolanic set of reactions. For this purpose, and in our first report, XRPD and µCT were used to analyse a PC paste-filled capillary (Salcedo *et al.*, 2021 ▸). That work established some experimental parameters but it did not carry out any time-dependent hydration study. In a second recent report (Shirani *et al.*, 2024 ▸), we extended this approach to study a hydrating cement, PC 42.5 R, at 1, 3, 7 and 77 d. The results of the analyses at 7 d within the hydrating capillary were compared with *ex situ* prepared pastes which were additionally studied by thermal analysis, calorimetry and XRPD. The results for the *ex situ* and *in situ* analyses were very similar, allowing us to establish the accuracy of the procedures. Here, we further broaden this methodology by studying the hydration of three related pastes: (i) another fine PC 52.5 R, and two blends of this cement with (ii) quartz and (iii) limestone. The obtained results are proven to be very robust and accurate. Portlandite easily carbonates but this methodology can measure it very reliably. Ettringite is very labile and the reported approach can measure it faithfully. These are necessary steps before extending this methodology to key PC–SCM blends, which are more challenging to analyse because of the extra difficulty of following the additional reactions during hydration. The final objective is to determine robustly the pozzolanic activity of SCMs by employing this combination of techniques.

## 2. Experimental

### 2.1. Materials

For this study, a CEM I 52.5 R commercial Portland cement was used that fulfils EN 197-1. Hereafter, this sample is abbreviated as PC-525. To prepare the corresponding blends, commercial quartz (Qz) and limestone (LS) were also employed. Qz was supplied by José Sanchís Penella, S.A. (Spain). LS was supplied by Omya Clariana S.L.U. (Spain) and its trademark is Omyacarb 12 Extra-PU.

The preparation of the two blends, (i) 80% of PC-525 and 20% of Qz, and (ii) 80% of PC-525 and 20% of LS, is detailed next. PC-525 (120 g) and Qz (or LS) (30 g) were weighed and subsequently introduced into a ∼1.3 l vessel with three steel balls of 33 mm in a Micro-Deval ball mill (Proeti). The mixtures were stirred at 100 rev min^−1^ for 1.5 h. After a 30 min resting period, the mixtures were stirred again for another 1.5 h. The blends are hereafter abbreviated as PC-20Qz and PC-20LS.

### 2.2. Paste preparation

The pastes were prepared using the same protocol for all measurements. Neat PC-525 (8.00 g) was weighed and mixed with twice-boiled distilled water (4.00 g) to give a water-to-cement mass ratio (w/c) of 0.50. For the blends, PC-20Qz (or PC-20LS) (8.00 g) and twice-distilled water (3.20 g) were mixed to yield a water-to-binder mass ratio (w/b) of 0.40. The mixtures were manually stirred for 60 s and then stirred in a vortex mixer for another 60 s. Boiled water was used to eliminate any dissolved CO_2_, and a plastic film covering was used to prevent CO_2_ diffusion during cooling.

For *in situ* LXRPD and µCT analyses, the pastes were syringed into 2.0 mm nominal (outer) diameter glass tubes, with a wall thickness of 0.01 mm. Using the *Dragonfly* software [version 2022.1 for *Windows*; Object Research Systems (ORS) Inc., Montreal, Canada], the internal diameter in the measured region was estimated to be approximately 1.7 mm. UV-curing adhesive was used to seal both ends to prevent carbonation and loss of water, as previously described (Shirani *et al.*, 2024 ▸). For the calorimetric studies, 6 g samples of the pastes described above were injected into the calorimeter glass ampoules.

### 2.3. Methods

#### 2.3.1. Textural characterization techniques

Particle size distributions (PSDs), and hence particle sizes, were measured by laser diffraction with a Mastersizer 3000 instrument (Malvern Panalytical) provided with a dry chamber (Aero S). The refractive and absorption indexes used were 1.68 and 0.1 for PC, 1.54 and 0.01 for quartz, and 1.66 and 0.01 for limestone, respectively. These values are provided by Malvern Panalytical in their software database, *Mastersizer-v3.81*. The specific surface area was measured by nitro­gen isotherm employing the BET approach using an ASAP 2420 instrument (Micromeritics, USA). The density was measured by He pycnometry using an Accupyc II 1320 pycnometer (Micromeritics).

#### 2.3.2. X-ray fluorescence

X-ray fluorescence data were measured in an ARL ADVANT’XP+ Thermo Fisher instrument. Samples were prepared in fused beads.

#### 2.3.3. Isothermal calorimetry

The analyses were performed in an eight-channel thermal activity monitor microcalorimeter from TA Instruments. Measurements were taken up to 7 d at 20°C, excluding the first 45 min after mixing to stabilize the equipment thermally.

#### 2.3.4. Laboratory X-ray powder diffraction

Transmission powder X-ray diffraction data were recorded using a Bruker D8 ADVANCE diffractometer with monochromatic Mo *K*α_1_ (λ = 0.7093 Å) radiation. The experimental setup has been previously reported (Shirani *et al.*, 2024 ▸). The powder diffraction patterns for the pastes were collected sequentially with the µCT data acquisition, measuring the same volume of the capillary.

#### 2.3.5. Laboratory X-ray computed microtomography

A Bruker SKYSCAN 2214 µCT system was employed to acquire the µCT data sets. As previously described (Shirani *et al.*, 2024 ▸), the capillaries were positioned in a custom-designed sample holder to scan the same volume with time (field of view 2.2 × 1.5 mm, horizontal × vertical). The samples were scanned using an LaB_6_ source filament operated at 55 kV and 130 µA. To reduce beam hardening artefacts, a 0.25 mm Al foil was positioned in front of the CCD3 detector (physical pixel size of 17.4 µm). A 1.1 µm voxel size was achieved by setting a sample-to-source distance of 9.953 mm and a sample-to-detector distance of 305.496 mm and employing 2 × 2 binning. A total of 1801 projections were taken over 360° (0.2° rotation step) with an exposure time of 1.9 s and three-frame averaging; hence the data set resulted in a total recording time of 225 min.

Images were reconstructed using the *NRecon* software (Version 2.2.0.6, Bruker) employing (i) the required post-alignment, (ii) 30% of beam hardening correction, (iii) smoothing = 1 with smoothing kernel = 2 (Gaussian), (iv) a minimum for CS (cross section) to image conversion of −0.045, −0.010 or −0.020, and (v) a maximum for CS to image conversion of 0.550, 0.550 and 0.700 for PC-525, PC-20Qz and PC-20LS, respectively.

Manual registration was required to align the different scans for adequate data analysis. The procedure is detailed elsewhere (Shirani *et al.*, 2024 ▸).

#### 2.3.6. X-ray powder diffraction data analysis

Analysis of the XRPD data was carried out by the Rietveld method with the *GSAS* suite of programs (Larson & Von Dreele, 2004 ▸). The pseudo-Voigt peak shape function was corrected to account for the observed axial asymmetry (Thompson *et al.*, 1987 ▸; Finger *et al.*, 1994 ▸). The overall varied parameters included background coefficients, zero-shift error/sample displacement, phase scale factors, unit-cell parameters and peak shape parameters, and preferred orientation when needed. The employed crystal structures are described elsewhere (De la Torre *et al.*, 2017 ▸; Aranda *et al.*, 2017 ▸; Shirani *et al.*, 2024 ▸). To make comparisons, the data must be referred to a constant basis. In this approach, the results are referred to 100 g of paste, as explained below. When referring to a constant base, it is easy to calculate the degree of hydration for each component.

#### 2.3.7. Cement paste phase content normalization

The procedure described here is intended to estimate the overall quantity of amorphous phases in the hydrating cement paste in order to refer the analyses to a constant basis, *i.e.* 100 g of cement/binder paste. This approach does not require internal or external standards, as it is based on the hydration reactions that take place during cement hydration. However, it does require knowledge of the stoichiometries of the reactions yielding the amorphous phases. The amounts of the different amorphous phases are calculated from the reactions of the corresponding crystalline phases (Shirani *et al.*, 2024 ▸). The procedure has been recently sketched out (Shirani *et al.*, 2024 ▸) and it is thoroughly described here. Table 1 ▸ displays the hydration reactions that can take place in neat Portland cement hydration, *i.e.* not including any pozzolanic hydration reaction(s).

**Figure 1:**
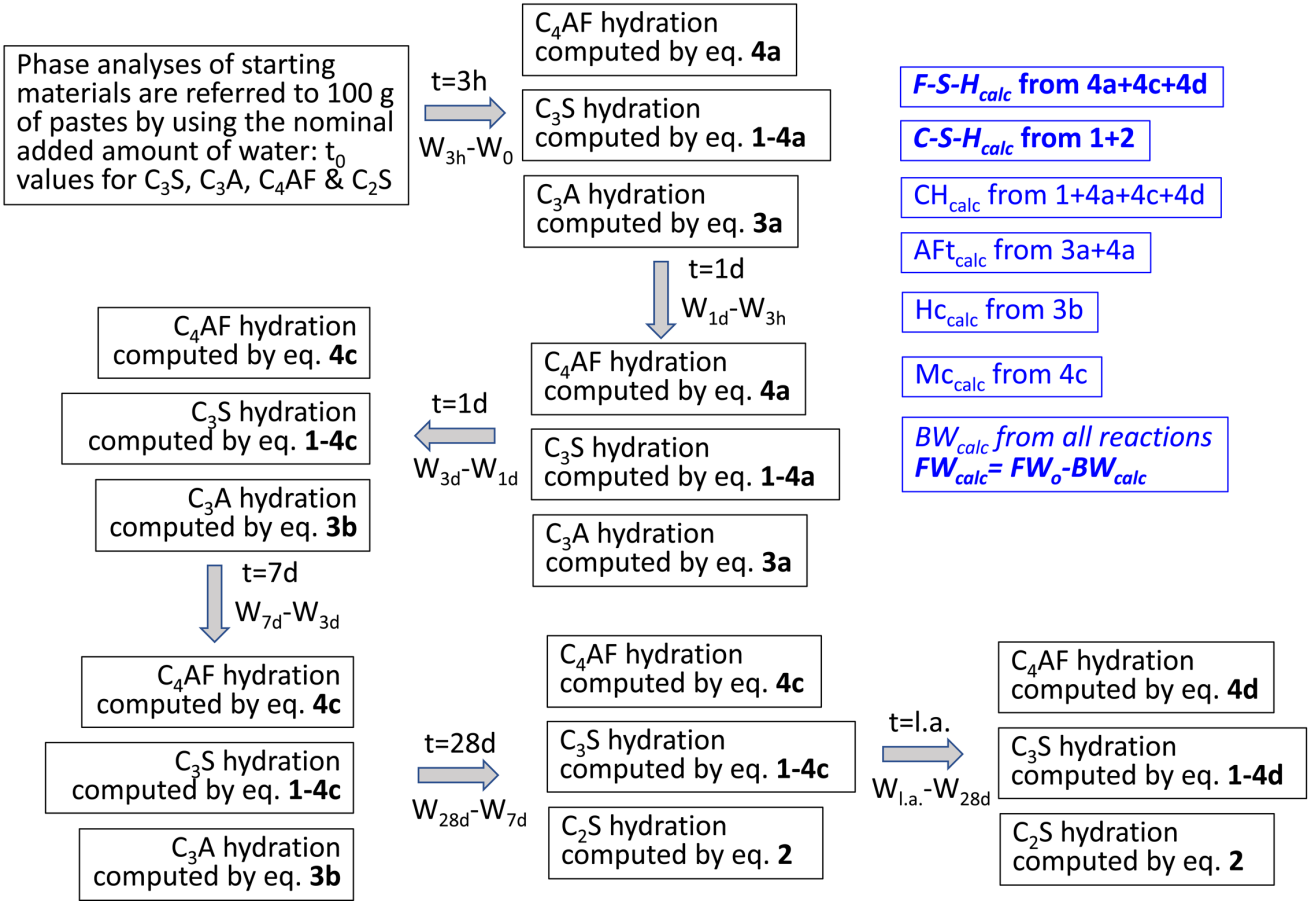
A flowchart with the steps followed for calculation of the amounts of hydrated phases, including amorphous ones (highlighted in italics). The stoichiometries of the hydration reactions are given in Table 1.

The calculated amounts of hydrated phases (crystalline and amorphous) were obtained by the hydration equations given in Table 1 ▸ considering the amounts of the reactants. The steps for the calculations are shown graphically in Fig. 1 ▸ and are based on previous publications (Avet *et al.*, 2018 ▸; Huang *et al.*, 2021 ▸; Briki, Avet *et al.*, 2021 ▸; Shirani *et al.*, 2024 ▸), but they are adapted here. Initially, the phase contents of the anhydrous binders are referred to 100 g of paste. For the PC-525 paste, since the w/c ratio was 0.50, the normalization factor was 0.667, *i.e.* 100/150. For the other two pastes, the employed w/b ratio was 0.40 and therefore the rescaling factor was 0.714. In a second step, the reacted amounts in a given time interval, for instance from initial mixing (*t* = 0) to 3 h, are determined by subtraction. Because of the existence of experimental errors, if the amount of a given phase is (slightly) larger at a later hydration age, the result of this subtraction is set to 0.

In the 0–3 h time period, the reacted crystalline C_4_AF fraction is computed according to equation (4*a*). This yields calculated amounts of amorphous iron–siliceous hydro­garnet C_3_FS_0.84_H_4.32_ (Fe–Si–H), AFt and CH. The stoichiometry assumed for Fe–Si–H was initially reported by Dilnesa *et al.* (2014 ▸) and it is being widely adopted (Avet *et al.*, 2018 ▸; Shirani *et al.*, 2021 ▸; Zunino *et al.*, 2022 ▸). The reacted amount of C_3_S, after subtraction of the C_3_S required for equation (4*a*), is computed according to equation (1). This yields calculated amounts of C–S–H and CH. The assumed stoichiometry for C–S–H gel is (CaO)_1.8_SiO_2_(H_2_O)_4.0_, which includes the gel pore water. The C/S ratio in C–S–H/C–A–S–H gels is variable but for the hydration of neat PC pastes is close to 1.8 (Zhu & Richardson, 2023 ▸; Cuesta *et al.*, 2018 ▸). The total amount of CH is obtained by equations (4*a*) and (1). At this time interval, the reacted C_3_A fraction is computed according to equation (3*a*). This yields a calculated value of AFt which is added to the one resulting from C_4_AF hydration.

In the 3 h to 1 d time period, because AFt keeps increasing and Hc is not formed, the same equations detailed above are applied. In the 1–3 d time period, AFt does not significantly increase and Hc and Mc are formed. Therefore, different chemical reactions are applied for C_3_A and C_4_AF. The C_4_AF fraction is computed according to equation (4*c*). This yields calculated amounts of amorphous Fe–Si–H, Mc and CH. The reacted C_3_S content, after subtraction of the C_3_S required for equation (4*c*), is computed according to equation (1). The reacted C_3_A fraction is computed according to equation (3*b*), yielding Hc. In the 3–7 d time period, exactly the same equations detailed for the previous time interval are applied. In the 7–28 d time interval, C_3_A reaction is not considered as its content at 7 d is negligible, but C_2_S starts to hydrate which should be considered. Therefore, the C_4_AF fraction is computed according to equation (4*c*), C_3_S is computed according to equation (1) and the C_2_S reacted fraction is considered by equation (2). At later ages, the hydration of C_3_S and C_2_S is considered with equations (1) and (2), respectively. However, at later ages, C_4_AF is considered to react according to equation (4*d*) which is a modified version where the iron–siliceous hydro­garnet also contains aluminium. This approach can be adapted to the different binders depending upon the consumption of the reactants and the formation of the crystalline products.

The procedure sketched above has been implemented in an *Excel* (Microsoft) file allowing us to refer the results to 100 g of paste. In order to do so, several assumptions are made.

(i) The (possible) amorphous contents of the initial binders are not considered and the amorphous content of the pastes is just the added amounts of water.

(ii) The amorphous content at a given time is the sum of the calculated amorphous components, C–S–H gel, Fe–Si–H and FW. FW is calculated as the amount of nominal (added) water minus the amount of chemically bound water, taking into account the chemical hydration reactions listed in Table 1 ▸.

(iii) Other (possible) amorphous phases, for instance, AFm-type, are neglected. The assumption of no amorphous AFm content is an approximation but is in line with recent findings showing that the reacted Al content is mainly within crystalline AFt, Hc and Mc and incorporated within C–S–H (Hemstad *et al.*, 2024 ▸).

With these three considerations, the normalization factor is iteratively varied, starting with the value obtained in the previous hydration time, until the best possible agreement is achieved between the measured CH value and the calculated one after applying the normalization. The results for unreactive phases such as quartz, calcite and belite (at early ages) allow us to check the procedure.

Importantly, because C–S–H and Fe–Si–H phases are formed, the overall amount of amorphous phases keeps smoothly increasing with hydration time. In other words, the normalization factor, *i.e.* 0.667 for PC-525 paste, decreases with time. There are other cements, *e.g.* calcium sulfoalumin­ate cements, where ye’elimite reacts with water to yield ettringite and minor amounts of amorphous aluminium hydroxide. In this particular case, the mass of the crystalline products is larger than that of the reactants and this normalization factor increases with time. This type of cement is being currently studied and the results will be reported elsewhere.

## 3. Results and discussion

This section is structured as follows. In the first subsection, the initial characterization of the employed materials is reported. This subsection also reports the calorimetric study of the pastes and the continuous study of the powder diffraction patterns for the PC-20Qz paste for about 3 d. In the following three subsections, the results for the *in situ* XRPD studies of PC-525, PC-20Qz and PC-20LS are presented and discussed. This characterization includes the calculated amounts based on the hydration reactions given in Table 1 ▸. The *in situ* µCT studies for the pastes are then reported, *i.e.* including the results previously obtained for PC-425.

### 3.1. Initial characterization

**Figure 2:**
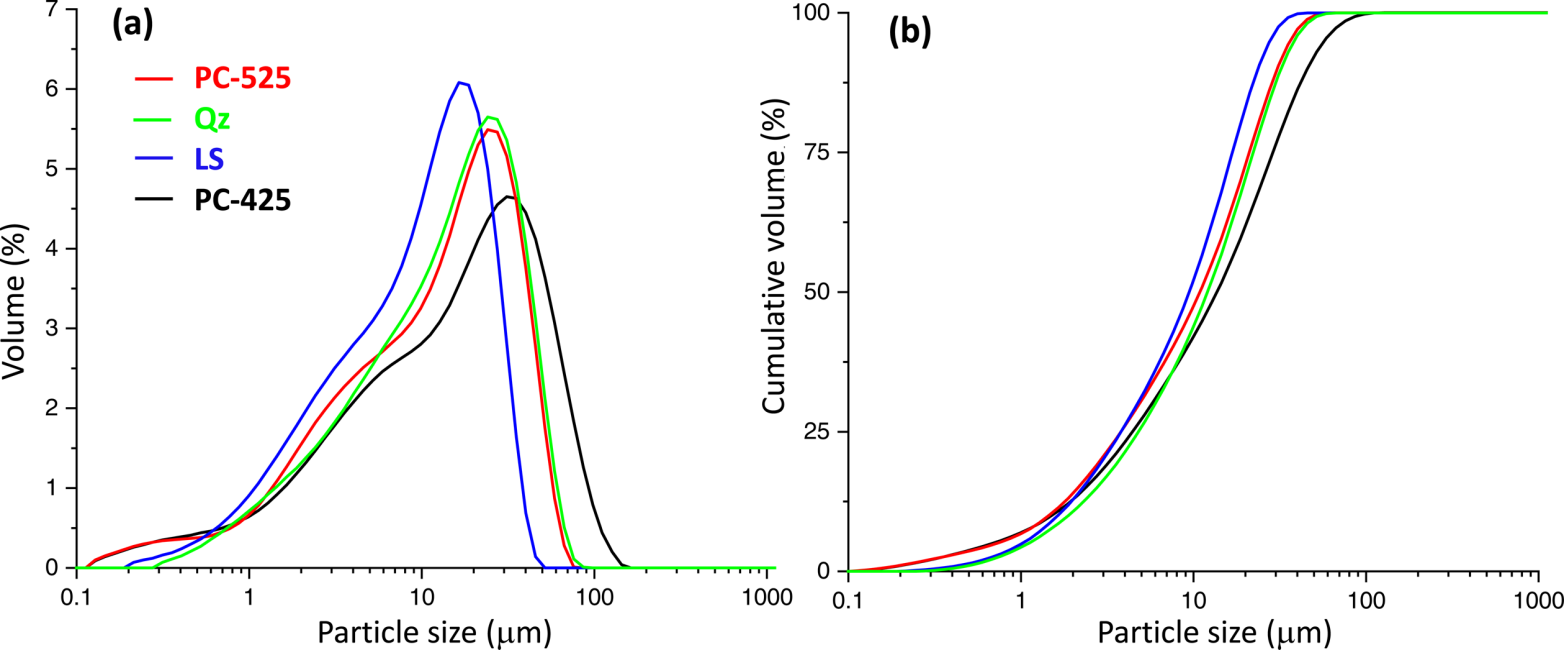
Particle size distribution for the studied starting materials. (*a*) Relative volume percentage. (*b*) Cumulative volume.

The elemental compositions of the materials used here are given in Table 2 ▸. This table also displays the values previously published (Shirani *et al.*, 2024 ▸) for PC-425, for easy access to the information. The corresponding mineralogical compositions are given in Table 3 ▸. Potassium sulfates, single or double salts, were not considered as their diffraction peaks are very broad and severely overlap. Microstructural properties are as important as the elemental and mineralogical compositions to understand the reactivity of cements. Therefore, Fig. 2 ▸ displays the PSD traces, and Table 4 ▸ gathers the corresponding values and the specific surface areas and Blaine fineness. The particle sizes of quartz and limestone are quite similar to those of PC-525.

**Figure 3:**
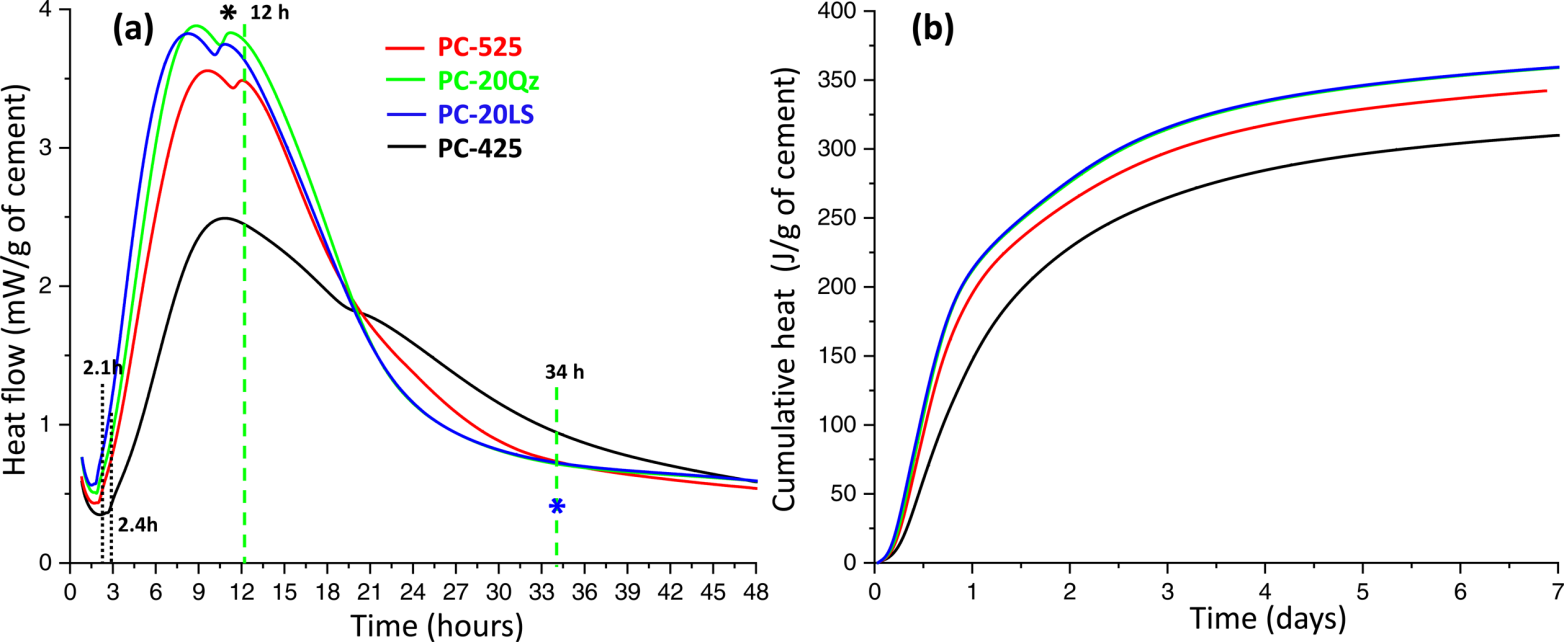
Isothermal calorimetry traces for the pastes at 20°C for up to 7 d. (*a*) Heat flow. (*b*) Cumulative heat. The results are referred to 1 g of cement. The ends of the induction periods for PC-525 and PC-425 are also displayed. The dashed lines at 12 and 34 h are explained in the text.

The overall hydration kinetics of the studied pastes have been investigated by isothermal calorimetry (Fig. 3 ▸). Fig. 3 ▸(*a*) displays the heat flows for up to 2 d, for better visualization, while Fig. 3 ▸(*b*) shows the cumulative heat up to the final time of the measurements, *i.e.* one week. The released heats at 7 d of hydration were 342.2, 359.0, 359.3 and 310.0 J g^−1^ of cement for PC-525, PC-20Qz, PC-20LS and PC-425, respectively. The results are totally in line with cement chemistry knowledge (Taylor, 1997 ▸; Scrivener *et al.*, 2016 ▸). PC-525 releases more heat and is faster than PC-425, mainly because of the smaller particle sizes of its constituents. Cements PC-20Qz and PC-20LS yield more heat and are faster than PC-525, as expected because of the filler effect (Oey *et al.*, 2013 ▸; Berodier & Scrivener, 2014 ▸; Kumar *et al.*, 2017 ▸). The additional surface available, due to the added quartz or calcite, mainly promotes the heterogeneous nucleation and growth of C–S–H gel from alite hydration.

**Figure 4:**
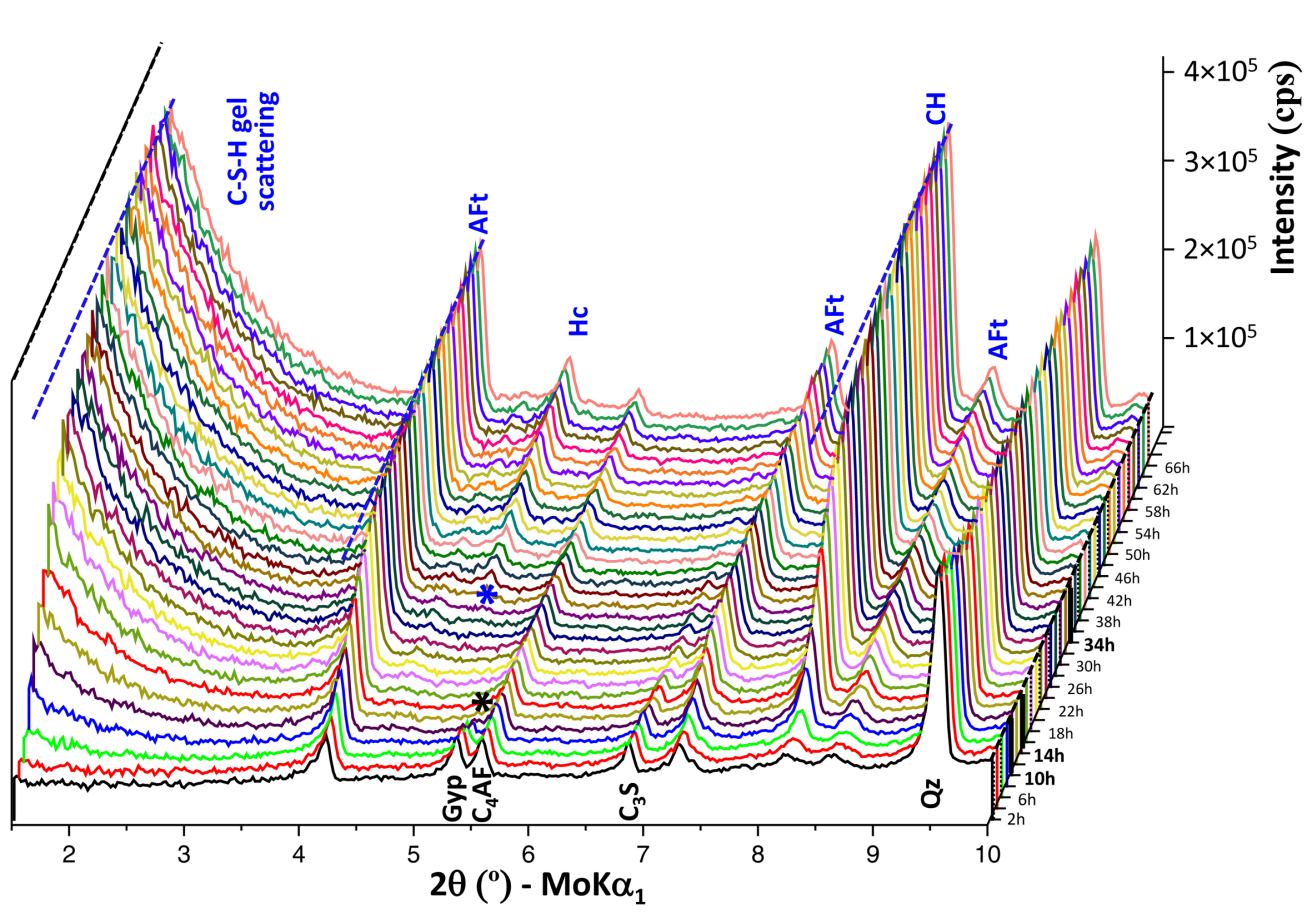
A selected 3D view between 1.5 and 10° 2θ of the laboratory Mo *K*α_1_ XRPD patterns for the PC-20Qz paste, w/b = 0.40. The positions of the main diffraction peaks corresponding to the anhydrous cement phases are labelled in black. The employed addition, quartz, is also labelled in black. The positions of the peaks due to crystallization of the hydrated phases are highlighted in blue.

An initial XRPD study was performed for the PC-20Qz paste, which was repeatedly scanned after water mixing for 66 h. This work was carried out in addition to the powder patterns collected for the joint Rietveld and µCT study discussed below. The paste was the same but the capillary was different. Fig. 4 ▸ displays a 3D view of all the patterns over a selected 2θ range, *i.e.* 1.5 to 10° 2θ (Mo *K*α_1_ radiation), for better visualization. Related to the unhydrated cement phases, gypsum is fully dissolved at ∼12–14 h [dashed line in Fig. 3 ▸(*a*)] after water mixing. This is the time where the overlapped peak (shoulder) in the calorimetry signal is observed [Fig. 3 ▸(*a*)]. This peak is usually named the sulfate depletion peak and it signals a reduction in the sulfate concentration in the pore solution. Fig. 4 ▸ demonstrates that crystalline AFm-type phases are not formed at this hydration time.

Concerning formation of the crystalline hydrated phase, several conclusions can be drawn.

(i) The powder diffraction peaks of AFt are already present during the first powder pattern collected at 2 h because the hydration reaction described by equation (3*a*) is very fast.

(ii) The integrated intensities of AFt grow rapidly at early ages, *i.e.* during the first 24 h.

(iii) AFt crystallization keeps occurring well after full gypsum dissolution at ∼14 h. This has been repeatedly reported (Jansen *et al.*, 2018 ▸; Bérodier *et al.*, 2020 ▸; Morales-Cantero *et al.*, 2024 ▸). The necessary sulfates come from desorption from the C–S–H gel.

(iv) CH diffraction peaks have low intensity in the first 4 h and their intensities start to increase rapidly after the end of the induction period.

(v) Hc diffraction peaks start to appear at ∼34 h [dashed line in Fig. 3 ▸(*a*)]. This has no clear associated thermal effect in the calorimetry signal [Fig. 3 ▸(*a*)]. However, detailed inspection shows a slight bump upwards compared with the smooth descending behaviour in the 15–21 h time interval; this bump is often related to AFm precipitation.

(vi) Finally, an increase in the low-angle scattering due to the precipitation of nearly amorphous C–S–H gel, located in the 1.5–2.5° range, is measured after 4 h, when the induction period ends.

### 3.2. *In situ* XRPD study for PC-525

**Figure 5:**
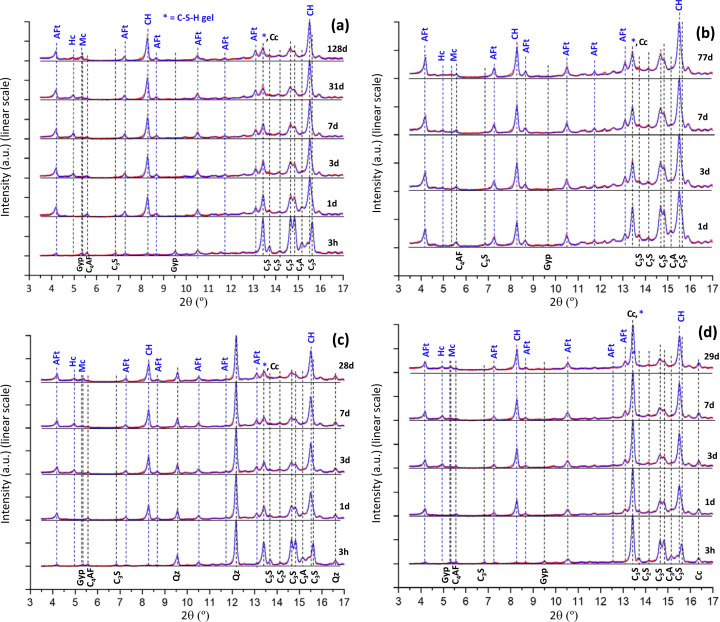
Mo *K*α_1_ Rietveld plots for the studied pastes displayed on a linear scale; red points indicate measured patterns and blue lines are calculated patterns. The difference curves are available but are not shown for the sake of clarity. (*a*) PC-525. (*b*) PC-425. (*c*) PC-20Qz. (*d*) PC-20LS. The positions of the main diffraction peaks corresponding to the (disappearing) anhydrous cement phases are labelled in black and highlighted with black dashed lines. The positions of the main diffraction peaks corresponding to the (appearing) hydrated cement phases are labelled in blue and highlighted with blue dashed lines. The patterns are vertically displaced for better visualization. Data for PC-425 are replotted from the results given in the original publication (Shirani *et al.*, 2024 ▸).

**Figure 6:**
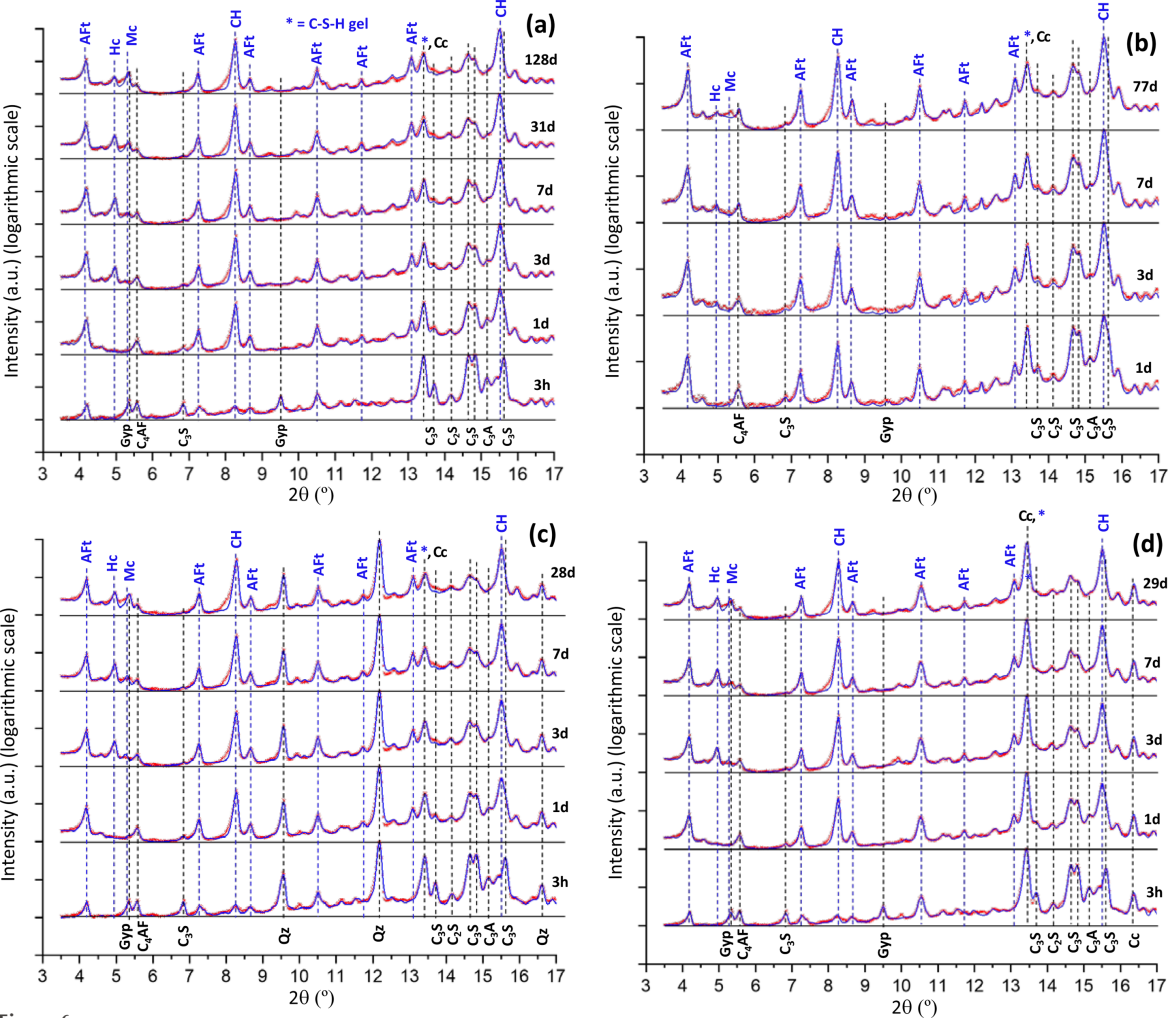
Mo *K*α_1_ Rietveld plots for the studied pastes displayed on a logarithmic scale. (*a*) PC-525. (*b*) PC-425. (*c*) PC-20Qz. (*d*) PC-20LS. All other details are as given in Fig. 5.

XRPD patterns for PC-525 paste were collected at six hydration ages. The Rietveld analyses were carried out as described in the *Experimental* section[Sec sec2]. The resulting Rietveld plots are displayed on a linear scale in Fig. 5 ▸(*a*) and on a logarithmic scale in Fig. 6 ▸(*a*). Displaying the Rietveld plots on a logarithmic scale allows us to follow low-intensity/scattering features which are more difficult to observe on a linear scale. Additional scattering is measured between 4.5 and 6° (2θ/Mo *K*α_1_) at 3 d and afterwards. This signals the precipitation of poorly crystalline AFm-type phases. This precipitation takes place in addition to the crystallization of the Hc (hemicarbonate) and Mc (monocarbonate) phases. Additional scattering (*i.e.* background increase) is also observed between 13 and 16° (2θ/Mo *K*α_1_). This scattering is due to the precipitation of C–S–H gel. Finally, note that the portlandite peak at 8.5° (2θ/Mo *K*α_1_) is not perfectly fitted, but additional diffraction is observed in the low-angle region of these peaks. This is very likely due to the presence of crystallization defects in layered Ca(OH)_2_, which has been previously reported (Chaix-Pluchery *et al.*, 1983 ▸; Madeja *et al.*, 2023 ▸).

The Rietveld quantitative phase results, without any normalization procedure, are given in Table 5 ▸. They are not referred to a constant basis and therefore they need elaboration. The normalization procedure, from the raw results to the data referred to 100 g of paste, has been carried out as detailed in Section 2.3.7. The results referred to 100 g of paste are displayed in Table 6 ▸. At *t*_0_ the normalization factor is the nominal amount of cement in the paste, *i.e.* 0.667. As the hydration reactions progress, the amount of amorphous material increases and therefore this normalization factor (smoothly) decreases (Table 6 ▸). At 7 d of hydration the measured amount of portlandite is 14.4 wt%, which is slightly larger than that measured at the same age for PC-425 (12.5 wt%; Shirani *et al.*, 2024 ▸). This was expected as PC-525 is slightly more reactive due to its smaller particle sizes [see also Fig. 3 ▸(*b*)]. This comparison shows the robustness of the reported experimental procedure. Concerning the ettringite phase, Table 6 ▸ shows that the maximum measured amount was 8.7 wt%. As expected, this value is smaller than that for PC-425, *i.e.* 11 wt%, because the SO_3_ contents for PC-525 and PC-425 are 3.3 and 3.9%, respectively. The maximum amount of AFt, considering SO_4_^2−^ as the limiting reactant, would be 10.8 wt% and this value is never reached (Table 6 ▸). The higher calculated values for AFt from C_3_A dissolution (Table 6 ▸) mean that after 1 d the aluminates are not the limiting reactant. Therefore, AFm-type phases (amorphous and/or crystalline) should precipitate. Concerning Hc and Mc phases, larger amounts of Hc component are measured up to 7 d. At 31 d and later, the situation reverses and larger amounts are measured for Mc, the thermodynamically stable phase. This behaviour has been repeatedly reported in the literature in many cement systems, not only for neat Portland cements (Georget *et al.*, 2022 ▸). Quantitatively, this study shows that a small amount of amorphous AFm-type phase(s) should precipitate, which agrees with the qualitative observation of an increase in background scattering at 4.5–6° (2θ/Mo *K*α_1_). In this context, it must be noted that recent thermodynamic work (Lothenbach *et al.*, 2019 ▸) indicates that iron–siliceous hydro­garnet is the stable phase, in preference to AFm, under many conditions. Therefore, partial Al substitution within iron–siliceous hydro­garnet could be expected, which would lead to lower contents of (Al-rich) AFm-type phases.

The hydration degree of every clinker phase can be readily obtained from the data in Table 6 ▸ at the studied hydration ages as their contents are referred to a constant basis. These values will be considered for all the studied binders in the *General discussion* section[Sec sec4]. It is clarified that the crystalline C_3_A contents at 0 and 3 h were measured as 3.2 and 3.4 wt%, respectively. We interpret this as the variability inherent in any experimental result and not due to an actual increase in C_3_A amount. Hydration between 31 and 128 d is proved, as the alite and belite contents decrease and the portlandite content increases. This shows that hydration does not stop because of the self-desiccation effect (Wyrzykowski & Lura, 2016 ▸) with the employed experimental setup, *i.e.* a capillary with a thickness of 2 mm.

### 3.3. *In situ* XRPD study for PC-20Qz

Five Mo *K*α_1_ XRPD patterns were collected for the PC-20Qz paste. The Rietveld plots are displayed on a linear scale in Fig. 5 ▸(*c*) and on a logarithmic scale in Fig. 6 ▸(*c*). As discussed above, additional scattering due to poorly crystalline AFm phases is observed between 4.5 and 6° (2θ/Mo *K*α_1_) at 3 d and afterwards. C–S–H gel precipitation is also detected at 1 d and later ages as the background increases at 3.5° (2θ/Mo *K*α_1_), and also between 13 and 16° (2θ/Mo *K*α_1_).

The direct Rietveld results are given in Table 7 ▸ and the component percentages, referred to 100 g of paste, are gathered in Table 8 ▸. At *t*_0_ the normalization factor is the nominal amount of binder in the paste, *i.e.* 0.714. The w/b = 0.40 used corresponds to a water/cement phase mass ratio of 0.50, *i.e.* 40 g of water for 80 g of pristine cement. Hence, a possible difference in reactivity will not arise from a different w/c ratio with respect to PC-525 phases. As expected and discussed previously, the relative amount of amorphous phases increases with hydration time and therefore the normalization factor decreases smoothly with time. The measured amount of portlandite at 7 d is 12.7 wt%, which is slightly larger than that measured for PC-525 after 20% dilution, *i.e.* 11.5 wt%. This is very likely due to the enhanced reactivity of C_3_S because of the filler effect, as discussed in the calorimetric study in Section 3.1. This comparison shows again the high accuracy and robustness of the reported experimental procedure. Concerning the ettringite phase, the results are also very robust: 80% of the maximum amount of AFt measured for PC-525 would be 7.0 wt%, which agrees very well with the maximum amount of AFt measured for PC-20Qz, 6.9 wt% (Table 8 ▸). The evolution of the contents of Hc and Mc in PC-20Qz is totally in line with the values obtained for PC-525.

Quartz is an unreactive phase. Therefore, if the normalization procedure is correct the obtained values should be constant. This is indeed the case, and the measured values between 3 h and 28 d are in the range 15.1–15.4 wt%. The *t*_0_ value of 14.4 wt% is the nominal (weighed) one, considering that the cement has no amorphous or unaccounted phases. We considered this comparison quite satisfactory and we speculate that the small difference between 14.4 and 15.2 wt% is mainly due to the employed assumption of no amorphous phases in PC. Portland clinker could have a small fraction of subcooled (non-crystalline) liquid. It is acknowledged that the presence of non-diffracting phase(s) in grey Portland clinkers is debatable, with reports both for (Aranda *et al.*, 2012 ▸; Christidis *et al.*, 2021 ▸; Kang *et al.*, 2023 ▸) and against (Jansen, Stabler *et al.*, 2011 ▸; Snellings *et al.*, 2014 ▸). Additionally, the clinker is milled with calcium sulfate and limestone additions from quarries which are known to be of limited purity.

### 3.4. *In situ* XRPD study for PC-20LS

Five Mo *K*α_1_ XRPD patterns were also collected for the PC-20LS paste. The Rietveld plots are displayed in Figs. 5 ▸(*d*) and 6 ▸(*d*). The direct results are given in Table 9 ▸ and the phase contents based on 100 g of paste are shown in Table 10 ▸. This paste also had a w/b ratio of 0.40 and therefore, at *t*_0_, the normalization factor is 0.714. The obtained values for this normalization factor with time are very similar to those determined for PC-20Qz, which agrees with the very similar calorimetric traces (Fig. 3 ▸).

The amount of portlandite measured at 7 d for PC-20LS is 12.5 wt%. This value is the same, within error, as that measured for PC-20Qz, *i.e.* 12.7 wt%. Concerning ettringite, the results are again very robust. The AFt measured content ranges from 6.6 to 6.8 wt% in the 1–7 d interval. For PC-20Qz during this period, the AFt measured content ranges from 6.5 to 6.9 wt%. Interestingly, the Hc and Mc contents for PC-20LS are the same, within error, as those measured for PC-20Qz. This highlights that the carbonate content of the pristine cement is enough for aluminate reactivity and that the additional limestone in this blend only has filler and dilutive effects.

Calcite is partly reactive, but the measured amounts of Hc and Mc require the dissolution of less than 0.40 g of calcite, referred to 100 g of paste. The measured values of calcite between 3 h and 28 d range from 18.5 to 18.8 wt%. The *t*_0_ value, 17.4 wt%, is again a nominal one, considering that the cement has no amorphous or un­accounted phases. We consider this comparison also satisfactory and we speculate that the small difference between ∼17.0 and 18.6 wt% is mainly due to the assumption that PC has no amorphous phase(s).

### 3.5. *In situ* µCT study

When there is sufficient contrast between the components and sufficient spatial resolution, the dissolution of anhydrous phases can be directly visualized by µCT. This technique is complementary to LXRPD as it could allow us to quantify the dissolution of amorphous particles if they could be dis­entangled. For *in situ* studies interrogating the same volume of a sample, there is a significant constraint in the data analysis because anhydrous particles can only decrease in volume. This could be quite helpful for advanced data analysis approaches. Unfortunately, conventional laboratory X-ray attenuation µCT is unable to detect features smaller than the spatial resolution, and the contrast between the different components is not always enough for accurate segmentation.

**Figure 7:**
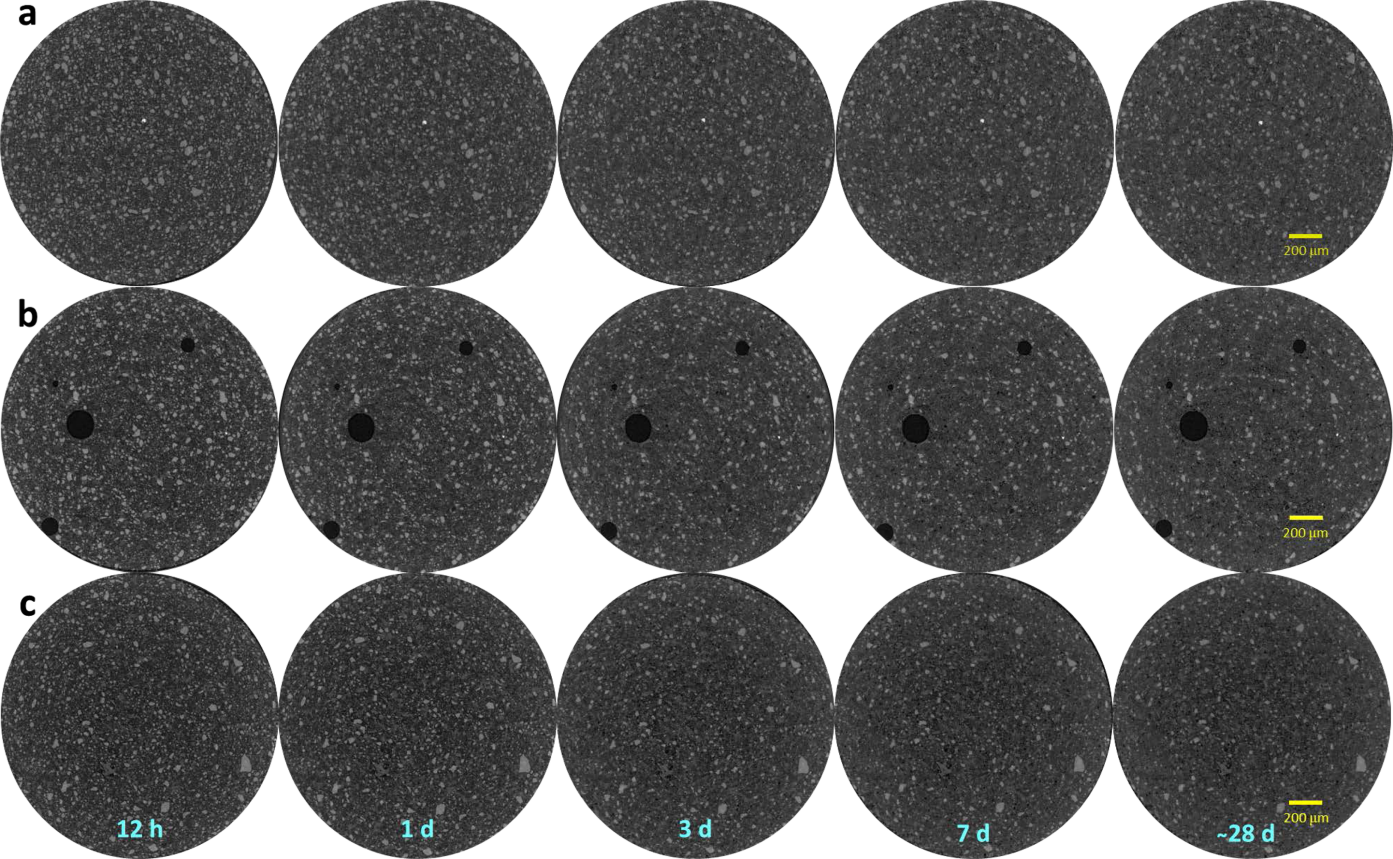
Selected µCT orthoslices at the studied hydration ages showing the overall hydration evolution. (*a*) PC-525. (*b*) PC-20Qz. (*c*) PC-20LS.

**Figure 8:**
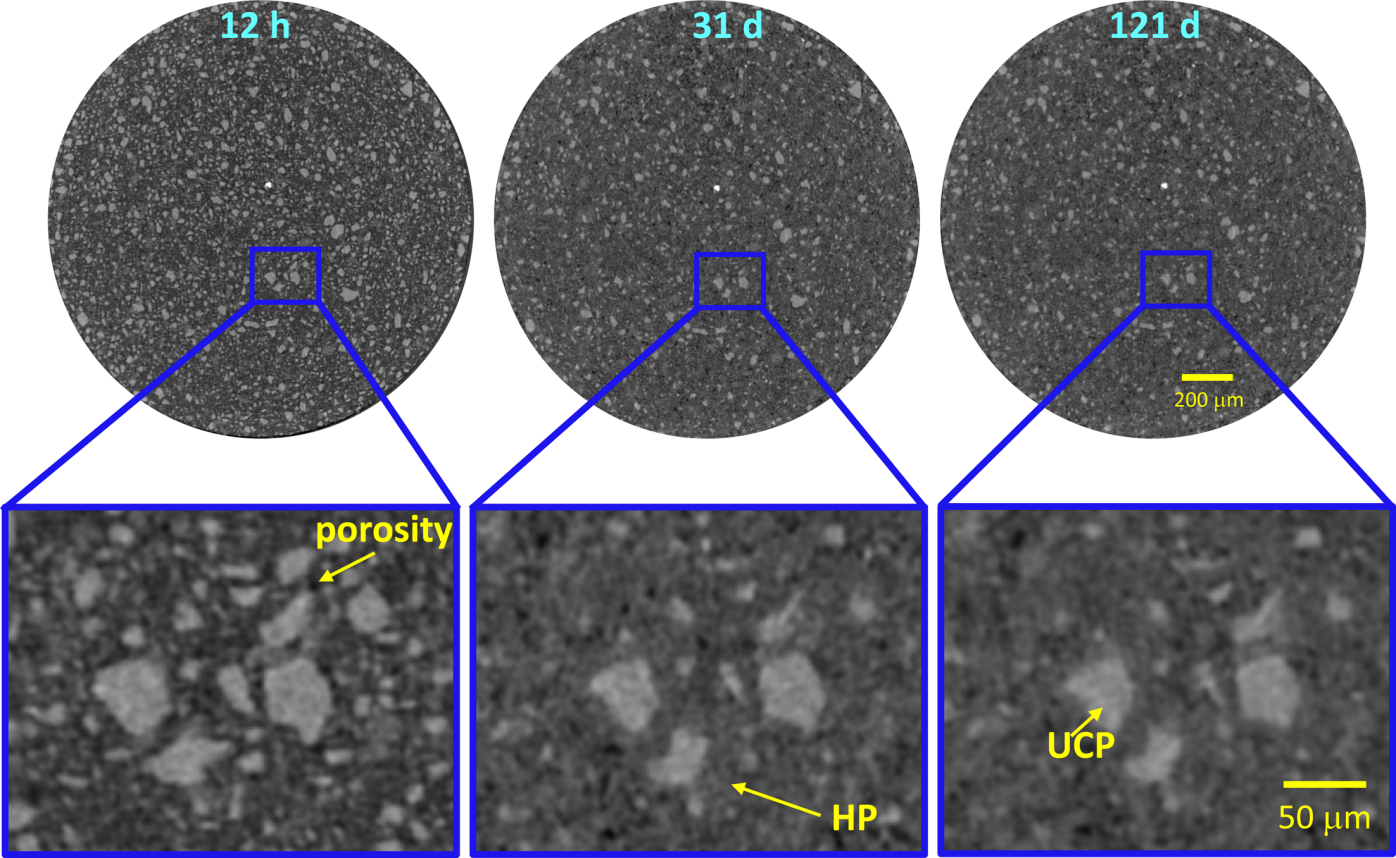
Selected µCT orthoslices for PC-525 at three hydration ages. (Top row) Full data showing the increased reactivity of small particles. (Bottom row) Enlarged views showing the changes in the paste as a function of hydration time and highlighting the three components that can be readily identified based on the grey values: porosity (darkest regions), HPs with intermediate grey values and UCPs (whitish particles).

With these caveats, Fig. 7 ▸ shows selected orthoslices for the studied pastes. The favoured dissolution of the smallest cement particles (brightest regions) is clearly noticeable, as is paste densification over time. PC-20Qz also shows tiny bubbles of entrained air. Fig. 8 ▸ displays enlarged views for PC-525, underlining the three components that can be easily identified: porosity (darkest regions), hydrated products (HPs, intermediate grey values) and unhydrated cement particles (UCPs, whitish particles). The HP regions include capillary water.

**Figure 9:**
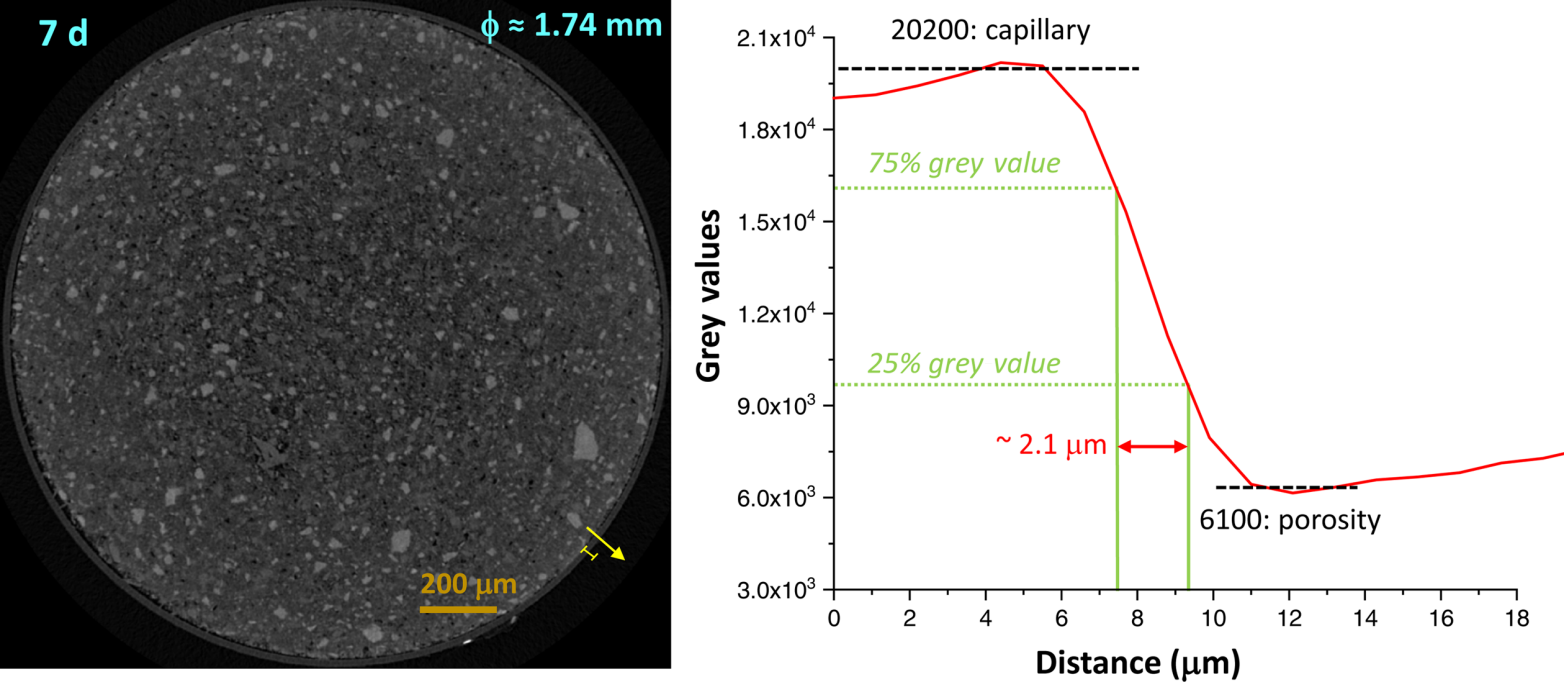
A µCT orthoslice for PC-525 at 7 d of hydration. The spatial resolution is estimated from the yellow line shown in the left-hand panel and the resulting grey-value plot is displayed in the right-hand panel (see text for details).

Pixel size is related to, but not the same as, spatial resolution. Currently, there is no general method for quantifying and reporting the resolution of a given tomogram. The spatial resolution was estimated by employing edge sharpness across interfaces. According to ISO/TS 24597 (Donnelly *et al.*, 2020 ▸), the resolution is defined as the Gaussian radius of the point spread function, which is the change between 25 and 75% grey value across the studied sharp interface (Donnelly *et al.*, 2020 ▸; Li *et al.*, 2023 ▸; Shirani *et al.*, 2023 ▸). This method, applied to the air/capillary outer wall boundary, gave 2.2 (3) µm from six measurements in three different capillaries. Fig. 9 ▸ displays one example of this type of plot. The obtained value for the spatial resolution is fully consistent with a former report (Shirani *et al.*, 2024 ▸). The spatial resolution, approximately 2.2 µm, has a significant impact. Particles smaller than this threshold cannot be identified and are instead considered part of a neighbouring component, which is likely to be the most abundant one, *i.e.* a hydrated phase. As demonstrated below, this feature results in a slight overestimation of HP and a small underestimation of the UCP fraction.

**Figure 10:**
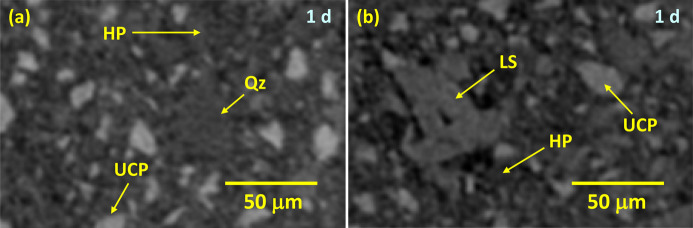
µCT enlarged views for (*a*) PC-20Qz and (*b*) PC-20LS. In addition to HP and UCP components, Qz particles in PC-20Qz and LS particles in PC-20LS are highlighted.

The PC–quartz blend has been employed here as a starting point for the PC–SCM blends to be investigated in a forthcoming report. Fig. 10 ▸(*a*) displays an enlarged view of an orthoslice of PC-20Qz showing quartz particles. The grey values of quartz are very similar to those shown by HPs, which makes its segmentation difficult as global thresholding cannot be employed. More sophisticated approaches, for instance using machine learning, are needed and this is currently being pursued. The PC–limestone blend was investigated as calcite is a very common addition in a range of low-carbon cements (Voglis *et al.*, 2005 ▸; Juenger *et al.*, 2019 ▸; Briki, Zajac *et al.*, 2021 ▸). Fig. 10 ▸(*b*) displays an enlarged view of an orthoslice of PC-20LS showing limestone particles. The grey values of calcite are slightly larger than those of HPs and lower than those of UCPs. This makes direct quantification of limestone also challenging.

**Figure 11:**
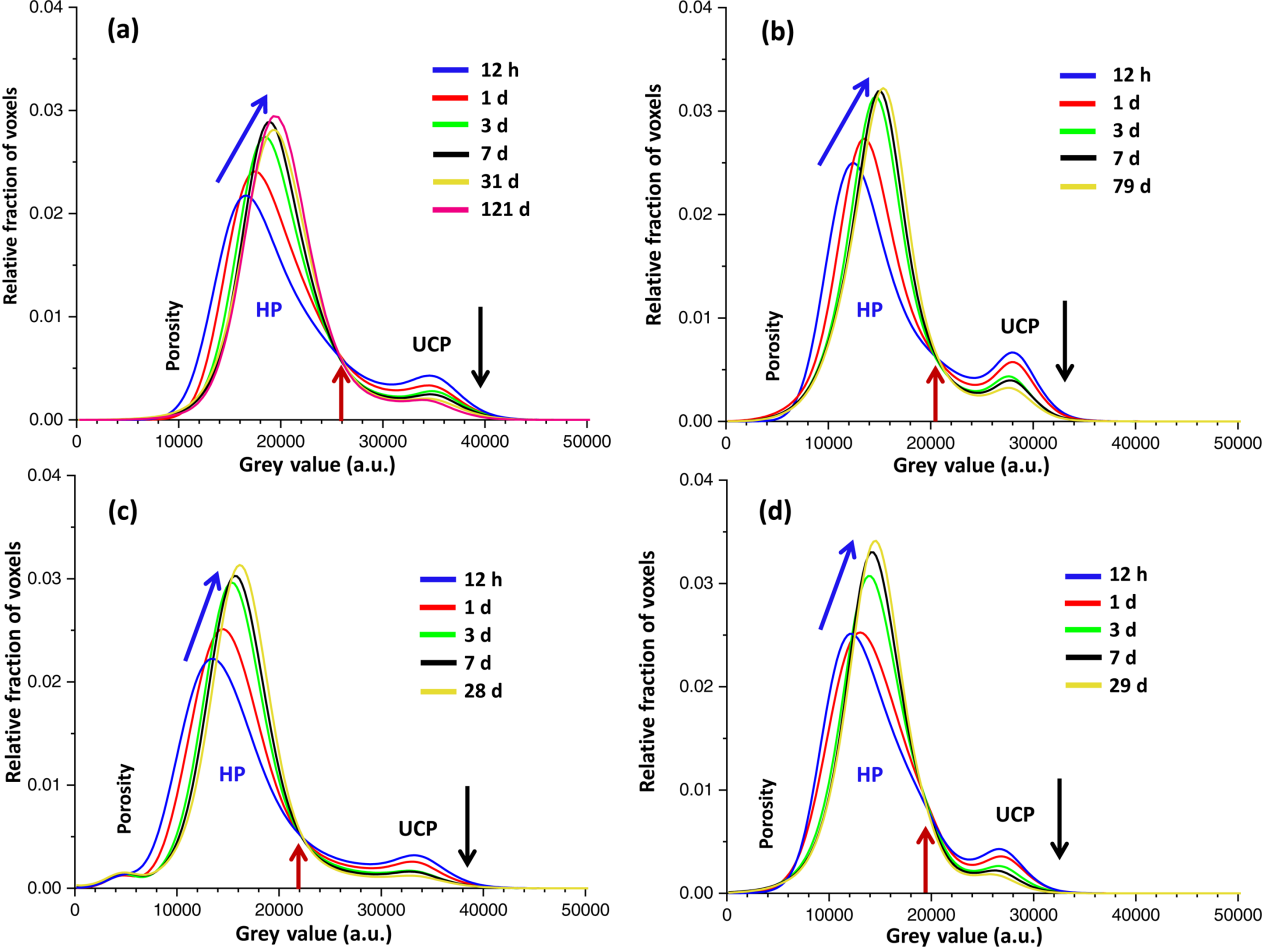
Time evolution of the grey-value histograms displaying the progress of the different components. (*a*) PC-525. (*b*) PC-425. (*c*) PC-20Qz. (*d*) PC-20LS. For a description of the labels, see the text.

A semiquantitative evaluation can be done on the basis of the time evolution of the grey-value histograms (Fig. 11 ▸). Several features merit discussion, being in line with our previous publication (Shirani *et al.*, 2024 ▸):

(i) The employed experimental setup permits separation of UCPs from HPs for particles larger than the spatial resolution. Nevertheless, partial volume effects cannot be avoided in cements as many particles are smaller than the spatial resolution (Aranda, 2016 ▸).

(ii) UCPs decrease over time for all pastes (as indicated by the black arrows in Fig. 11 ▸), while the amount of HP increases (as indicated by the blue arrows).

(iii) HPs densify with time and this is reflected by larger average grey values with hydration time (also blue arrows).

(iv) There are constant crossing points (brown arrows in Fig. 11 ▸) for all studied pastes. From the time evolutions, the particles with grey values above these thresholds are primarily UCPs, while particles with lower grey values are principally HPs.

(v) The signature of air porosity development, *i.e.* shrinkage and appearance of water-vapour-filled capillary pores, is evident in the left-hand tails of the HP bands.

(vi) As expected, the UCP/HP ratio is larger for PC-425 than for PC-525 because this last binder has smaller particle sizes which react faster. These ratios are even smaller for PC-20Qz and PC-20LS, as quartz and calcite have grey values within the HP region.

(vii) Quartz is not apparent in the histogram trace for PC-20Qz as its grey values are located at ∼14 000, which completely overlaps with the HP grey values.

(viii) Finally, calcite is barely seen in the histogram trace for PC-20LS, as a shoulder in the right-hand part of the HP bands with grey values very close to the crossing point for this paste.

**Figure 12:**
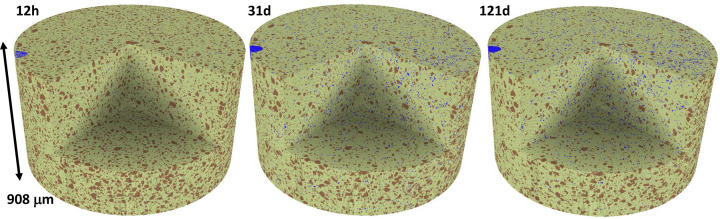
Four-dimensional renderings of the manual global thresholding segmentation output for PC-525 at 12 h, 31 d and 121 d of hydration. Colour code: porosity is shown in blue, hydration products in olive green and unhydrated cement particles in brown.

Quantitative analysis of the tomograms for PC-525, *i.e.* segmentation, has been carried out by manual global thresholding. PC-20Qz and PC-20LS CTs have not been segmented because the quartz and limestone components cannot be separated with this approach. The volume of interest (VOI) that was treated, ∼2.0 mm^3^, had a cylindrical shape with a height of approximately 0.9 mm and a diameter of around 1.7 mm, as shown in Fig. 12 ▸. The segmentation of the VOI using *Dragonfly* resulted in three components: UCPs, HPs and porosity. The grey value for the boundary between UCPs and HPs remained constant over time, with a 26 000 grey value for the experimental conditions used. In contrast, the HP/porosity boundary varies over time and was estimated for each data set using the tangent-slope approach (Shirani *et al.*, 2021 ▸). The grey values ranged from 10 000 to 14 000. Table 11 ▸ presents the volume percentages of the three components for PC-525 paste over time. Discussion of these results will be carried out in the next section, together with the RQPA output.

## 4. General discussion

The final goal of this ongoing research project is to establish a methodology that enables precise analysis of cement components over time from *in situ* powder diffraction and microtomography, avoiding any sample preparation step. It also seeks to avoid the use of internal standards as these may modify the kinetics because of the additional surface and possible release of ions. In our first report (Shirani *et al.*, 2024 ▸), PC 42.5 R was chosen because it has larger particles, *i.e.D*_*v*,50_ is 17.6 µm. Here, the research is expanded to investigate another cement and two additional blends. The second cement, PC 52.5 R, has a smaller particle size, *D*_*v*,50_ = 14.0 µm, and the paste also has w/c = 0.50. Two blends of 80 wt% PC 52.5 R and 20 wt% quartz (or calcite) have been studied in the form of pastes with w/b = 0.40. We note that fractions with particle sizes below the achieved spatial resolution of 2.2 µm, which are about 12 vol.% (Fig. 2 ▸), cannot be measured by µCT even if there is enough contrast for their identification.

To carry out the comparison of LXRPD–µCT results, firstly the RQPA contents given in the previous section, in wt%, must be transformed to vol.% considering the densities of the components (Balonis & Glasser, 2009 ▸). For this comparison, the clinker phases have been gathered, *i.e.* C_3_S, C_2_S, C_3_A and C_4_AF, within the overall UCP component. C–S–H gel comprises approximately 35 vol.% of the paste, expressed as (CaO)_1.8_SiO_2_(H_2_O)_4.0_, which includes the gel pore water but not the capillary water. Due to the intermixing of C–S–H and capillary water, when these two components are grouped they fill ∼60% of the volume. Table 11 ▸ reports the UCP/HP ratios with hydration time for PC-525 and PC-425. The UCP content comparison between *in situ* LXRPD and *in situ* µCT has to be cautiously carried out as the data are registered consecutively, *i.e.* with a small time difference. This could be relevant at 1 d, when the reactions are relatively fast, but is likely to be insignificant at 3 d or later. For PC-525, the agreement between UCP contents from LXRPD and µCT is within 1.3 vol.% at 1 d and within 2.5 vol.% at later hydration ages. This disagreement is considered acceptable taking into account the assumptions made, and indicates that the UCP results from µCT data are relatively accurate. As demonstrated by LXRPD and calorimetry, PC-425 is less reactive than PC-525. This is evident in Table 11 ▸ as, for a given age, the UCP contents for PC-425 are invariably larger than those for PC-525. Finally, note that µCT data for PC-20Qz and PC-20LS pastes have not been segmented as global thresholding does not allow the classification of quartz and calcite components. Machine learning segmentation is being tried and, if successful, it will be reported elsewhere.

This work uses absorption-based X-ray imaging, but X-ray grating-based imaging could help in the characterization of sub-resolution features. The implementation of these approaches is being extended from synchrotron beamlines to laboratory instruments (Prade *et al.*, 2016 ▸; Blykers *et al.*, 2022 ▸). X-ray diffraction/scattering computed tomography is also being extended to laboratory equipment (Cersoy *et al.*, 2015 ▸). However, the implementation of laboratory diffraction computed tomography in cement samples is really challenging because of the co-existence of high- and low-diffracting components.

Finally, it is appropriate to compare the developed LXRPD methodology and the obtained degree of hydration (DoH) results with previous publications. Concerning the LXRPD data collection method­ology, two approaches are usually followed in cement hydration studies. On the one hand, the pastes can be cast in plastic cylinders, sealed and cured until the required age. Immediately prior to the measurements, discs are cut and polished, usually with sandpaper (Lothenbach *et al.*, 2008 ▸; Durdziński *et al.*, 2017 ▸). On the other hand, the pastes can be cast in any container, sealed and cured. At a given hydration age, pieces (or ground powder) are immersed in an appropriate solvent (*e.g.* acetone or propan-2-ol) to arrest the hydration reactions. After solvent removal (under vacuum or by gentle heating), the powder is ground [see for instance Noguchi *et al.* (2021 ▸)]. This second approach gives good particle averaging for phases with larger grains, and portlandite is less susceptible to carbonation as free water is removed. It allows the use of additional techniques for the same powder, such as thermal analysis. However, labile components, *e.g.* ettringite, are partly destroyed in the hydration-arresting step. The first approach better preserves the labile phases, but the cutting and polishing steps require good experimental skills, and portlandite is easily carbonated. Neither of these two procedures allows for the same region to be analysed with time. This is an additional drawback for composite cements where there is a large inherent variability. The ‘mix and measure methodology’, very recently reported (Shirani *et al.*, 2024 ▸) and further developed here, does not affect labile phases and portlandite cannot be carbonated. It scans a large volume and allows measurement of the same region with time. A drawback is self-drying, although this is shown here not to affect capillaries with a diameter of 2 mm or wider. Of course, the main experimental limitation is the availability of Mo *K*α_1_ radiation.

Concerning the RQPA results, here it is shown that portlandite is not carbonated and that the AFt content does not decrease significantly even up to 128 d of hydration. Large errors in CH content determination have been reported in some cases for the paste disc methodology [see for instance Li & Scrivener (2022 ▸)] and very low AFt contents are frequently reported after hydration arresting [see for instance Elakneswaran *et al.* (2019 ▸)]. Because the data are referred to 100 g of paste, the calculation of the DoH at the measured hydration ages is straightforward. Table 12 ▸ displays the DoH of the clinker phases for the three studied pastes. The phase-dependent DoH can be compared with those published earlier but the comparison has to be exercised with care, as several features affect the DoH at a given time, including (i) the w/c ratio, (ii) the temperature of hydration, (iii) the fineness of the final cement, (iv) the alkali content and (v) the SO_3_ content.

Several conclusions can be drawn from the content of Table 12 ▸:

(i) C_3_S is the most reactive phase at early ages, *i.e.* less than 3 d. The DoH at 28 d is in the range 88–93% for the three studied pastes. These values compare very well with 88% reported by Lothenbach *et al.* (2008 ▸), on the basis of the work of Parrott & Killoh (1984 ▸), for a PC paste with w/c = 0.40. For a PC paste with w/c = 0.50, the C_3_S DoH was reported to be above 90% by Noguchi *et al.* (2021 ▸).

(ii) C_3_A reaches full hydration at 7 d. This is in very good agreement with 100% hydration after 7 d reported by Noguchi *et al.* (2021 ▸) and more than 85% hydration degree reported by Lothenbach *et al.* (2008 ▸).

(iii) The DoH for C_4_AF is 60–70% at 28 d. These values are a bit lower than those reported previously, 70 and 90% by Lothenbach *et al.* (2008 ▸) and Noguchi *et al.* (2021 ▸), respectively. C_4_AF reactivity varies significantly depending upon the melt composition and the clinkering thermal history (Peys *et al.*, 2022 ▸; Boháč *et al.*, 2024 ▸). It has been reported very recently that the C_4_AF hydration rate can be strongly enhanced by the additional surfaces of the SCMs (Redondo-Soto *et al.*, 2023 ▸; Morales-Cantero *et al.*, 2024 ▸) and accelerating admixtures (Peys *et al.*, 2022 ▸; Chang *et al.*, 2024 ▸) that can be present in the grinding agents.

(iv) As expected, C_2_S is the phase with the slowest hydration kinetics. Our results suggest that additional calcite may further slow down its hydration kinetics. For neat PC, we report about 20% DoH of belite at 28 d. Previous work reported about 50% DoH of belite at 28 d (Lothenbach *et al.*, 2008 ▸; Noguchi *et al.*, 2021 ▸), but we consider these values to be too high and they could be affected by the strong overlap of the C_2_S and C_3_S diffraction peaks.

(v) The filler effect (Oey *et al.*, 2013 ▸; Berodier & Scrivener, 2014 ▸; Kumar *et al.*, 2017 ▸) by quartz and limestone of moderate fineness, *D*_*v*,50_ = 15 and 11 µm, respectively, is firmly proved for C_3_S. The hydration kinetics of C_4_AF and C_3_A are also accelerated because of the presence of these additional surfaces.

## 5. Conclusions

This research extends an experimental protocol to study *in situ* cement hydration without any sample conditioning and avoids the use of an internal standard which would dilute the low-content phases even further. After water mixing, the pastes were syringed into 2.0 mm diameter glass capillaries whose ends were simply sealed with a polymer. The pastes underwent sequential analysis by X-ray microtomography and Mo *K*α_1_X-ray powder diffraction. The use of thick capillaries is crucial to prevent self-desiccation at later ages, which in turn is important to study pozzolanic materials. The sealing prevents portlandite carbonation which is an important source of errors in cement hydration studies. Other advantages of this approach, for powder diffraction, are excellent powder averaging and the minimization of preferred orientation. This protocol is tested here with a neat Portland cement type 52.5 R and two blends, 80% PC–20% quartz and 80% PC–20% limestone. Mass-balance calculations allow an estimation of the amount of amorphous phases and, with this, the Rietveld analysis results can be related to a constant basis, 100 g of paste.

The experimental procedure is shown to be robust and accurate. PC-525 has smaller particle sizes that react faster than PC-425. This is shown by powder diffraction and microtomography. The filler effect enhances the reactivity of clinker phases due to the presence of additional surfaces, and this has been quantitatively measured for the blends. The filler effect by quartz and limestone additions is quantified for alite, Ca_3_SiO_5_, and for the aluminate phases tricalcium aluminate, Ca_3_Al_2_O_6_, and brownmillerite, Ca_4_Al_2_Fe_2_O_10_. Chiefly, port­landite and ettringite phases are reliably quantified. This opens an avenue for quantifying the effect of admixtures (superplasticizers, accelerators, retarders *etc.*) with smaller experimental uncertainties. It will also allow the study of pozzolanic reactions at later hydration ages, *i.e.* between one and six months.

The microtomography data for PC-525 have been segmented and the comparison with RQPA results in an agreement within 2 vol.%. A crossing point in the histograms with hydration time for the three studied pastes is confirmed. This allows us accurately to disentangle the contributions from the clinker phases and from the hydrates. Unfortunately, the grey values for quartz and limestone do not allow segmentation by global thresholding because of severe overlapping.

## Figures and Tables

**Table 1:** Possible Portland cement hydration reactions

Hydration reaction	Equation
Ca_3_SiO_5_ + 5.2H_2_O → 1.2Ca(OH)_2_ + (CaO)_1.8_SiO_2_(H_2_O)_4.0_	(1)
Ca_2_SiO_4_ + 4.2H_2_O → 0.2Ca(OH)_2_ + (CaO)_1.8_SiO_2_(H_2_O)_4.0_	(2)
Ca_3_Al_2_O_6_ + 3CaSO_4_·2H_2_O + 26H_2_O → Ca_6_Al_2_(SO_4_)_3_(OH)_12_·26H_2_O	(3*a*)
Ca_3_Al_2_O_6_ + 0.5CaCO_3_ + 0.5Ca(OH)_2_ + 11.5H_2_O → Ca_4_Al_2_(OH)_13_(CO_3_)_0.5_(H_2_O)_5.5_	(3*b*)
Ca_3_Al_2_O_6_ + CaCO_3_ + 11H_2_O → Ca_4_Al_2_(OH)_12_(CO_3_)(H_2_O)_5_	(3*c*)
Ca_4_Al_2_Fe_2_O_10_ + 0.84Ca_3_SiO_5_ + 3CaSO_4_·2H_2_O + 30.84H_2_O → Ca_3_Fe_2_(SiO_4_)_0.84_(OH)_8.64_ + Ca_6_Al_2_(SO_4_)_3_(OH)_12_·26H_2_O + 0.52Ca(OH)_2_	(4*a*)
Ca_4_Al_2_Fe_2_O_10_ + 0.84Ca_3_SiO_5_ + 0.5CaCO_3_ + 16.34H_2_O → Ca_3_Fe_2_(SiO_4_)_0.84_(OH)_8.64_ + Ca_4_Al_2_(OH)_13_(CO_3_)_0.5_(H_2_O)_5.5_ + 0.02Ca(OH)_2_	(4*b*)
Ca_4_Al_2_Fe_2_O_10_ + 0.84Ca_3_SiO_5_ + CaCO_3_ + 15.84H_2_O → Ca_3_Fe_2_(SiO_4_)_0.84_(OH)_8.64_ + Ca_4_Al_2_(OH)_12_(CO_3_)(H_2_O)_5_ + 0.52Ca(OH)_2_	(4*c*)
Ca_4_Al_2_Fe_2_O_10_ + 1.68Ca_3_SiO_5_ + 11.68H_2_O → 2Ca_3_FeAl(SiO_4_)_0.84_(OH)_8.64_ + 3.04Ca(OH)_2_	(4*d*)

**Table 2:** Elemental analyses for PC 52.5R (PC-525), quartz (Qz), limestone (LS) and PC 42.5R (PC-425) All data are expressed as oxide weight percentage except for the loss on ignition (LOI).

	PC-525	Qz	LS	PC-425
CaO	64.2 (3)	0.074 (5)	56.0 (3)	61.6 (4)
SiO_2_	18.5 (3)	99.5 (3)		19.9 (3)
Al_2_O_3_	4.93 (9)		0.27 (3)	4.56 (9)
Fe_2_O_3_	3.0 (1)	0.039 (3)	0.050 (4)	3.3 (1)
SO_3_	3.3 (1)		0.040 (4)	3.9 (1)
MgO	1.2 (1)		0.83 (9)	1.5 (1)
K_2_O	0.64 (5)	0.081 (7)		1.14 (9)
Na_2_O	0.23 (5)			0.24 (5)
Others	0.5	0.02	0.03	0.7
LOI†	3.52	0.3	42.8	3.18

† Dried at 105°C and heated at 950°C for 2 h.

**Table 3:** Mineralogical compositions of the employed materials

Phases (wt%)	PC-525	Qz	LS	PC-425
C_3_S	64.0			58.3
C_2_S	9.5			12.9
C_3_A	4.8			6.7
C_4_AF	11.5			10.3
Cc	6.0		99.0	5.3
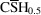				3.1
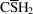	4.0			2.2
CaO				0.7
Qz	0.2	100.0		0.5
Dolomite			1.0	

**Table 4:** Microstructural and textural properties for the studied materials Specific surface area is abbreviated as s.s.a.

	PC-525	Qz	LS	PC-425
*D*_*v*,10_ (µm)	1.9 (1)	2.4 (1)	1.9 (1)	2.0 (1)
*D*_*v*,50_ (µm)	14.0 (2)	15.6 (6)	10.7 (1)	17.6 (1)
*D*_*v*,90_ (µm)	39.5 (1)	41.6 (1)	26.9 (1)	59.1 (5)
BET s.s.a. (m^2^ g^−1^)	1.37 (1)	0.93 (1)	1.04 (6)	1.88 (1)
Blaine fineness (m^2^ kg^−1^)	370			375
Density (g cm^−3^)	3.11 (1)	2.67 (1)	2.73 (1)	3.09 (1)

**Table 5:** Direct Rietveld quantitative phase analysis (RQPA) results (wt%) at different times for the PC-525 paste from *in situ* Mo *K*α_1_ XRPD

Phase	*t*_0_	3 h	1 d	3 d	7 d	31 d	128 d
C_3_S	64.0	61.7 (2)	25.2 (5)	16.0 (7)	15.5 (9)	12.7 (6)	11.2 (6)
C_2_S	9.5	9.1 (3)	12.7 (4)	14.3 (5)	14.7 (6)	12.1 (7)	11.7 (7)
C_3_A	4.8	5.2 (2)	3.5 (2)	1.5 (3)			
C_4_AF	11.5	10.3 (3)	11.7 (4)	9.4 (5)	8.6 (5)	7.2 (6)	6.2 (5)
Cc	6.0	4.7 (3)	7.6 (2)	7.7 (5)	4.9	5.7	7.8
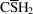	4.0	4.5 (2)					
Qz	0.2						
AFt		3.6 (2)	16.8 (3)	18.1 (4)	18.9 (4)	19.3 (5)	17.1 (5)
CH		1.1 (1)	22.5 (2)	29.0 (2)	31.2 (3)	34.7 (3)	36.9 (3)
Hc				2.8 (1)	3.8 (1)	3.3 (2)	1.8 (2)
Mc				1.3 (2)	2.3 (3)	5.0 (3)	7.2 (3)

**Table 6:** RQPA for the PC-525 paste (wt%) at different times from *in situ* Mo *K*α_1_ LXRPD (values referred to 100 g of paste) The calculated amounts of amorphous phases are given in italics. The calculated amounts of crystalline hydrated phases are also given for comparison purposes.

Phase	*t*_0_	3 h	1 d	3 d	7 d	31 d	128 d
C_3_S	42.7	40.6	12.9	7.6	7.2	5.3	4.4
C_2_S	6.3	6.0	6.5	6.8	6.8	5.0	4.6
C_3_A	3.2	3.4	1.8	0.7			
C_4_AF	7.7	6.8	6.0	4.5	4.0	3.0	2.5
Cc	4.0	3.1	3.9	3.7	2.3	2.3	3.1
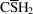	2.7	2.9					
Qz	0.1						
*H_2_O*	*33.3*	*31.3*	*16.9*	*13.8*	*12.5*	*10.7*	*10.6*
AFt/AFt_calc_†		2.4/2.3	8.6/10.8	8.6/10.8	8.7/10.8	8.0/10.8	6.8/10.8
CH/CH_calc_		0.7/0.7	11.5/11.5	13.7/13.3	14.4/13.3	14.4/14.2	14.6/14.7
H/Hc_calc_				1.3/2.3	1.8/3.8	1.4/3.8	0.7/3.8
Mc/Mc_calc_				0.6/1.8	1.1/2.3	2.1/3.5	2.8/3.3
*C–S–H_calc_*		*1.9*	*30.2*	*35.9*	*37.8*	*43.7*	*44.8*
*Fe–Si–H_calc_*		*0.8*	*1.6*	*3.0*	*3.5*	*4.4*	*5.1*
Factor	0.667	0.659	0.513	0.473	0.462	0.414	0.395

† The calculated amounts of ettringite, AFt_calc_, are invariably larger than the experimentally measured ones, AFt, because it is assumed that all aluminium from C_3_A and C_4_AF dissolution at 1 d yields ettringite. However, this is an approximation as it is known that about 20% of the aluminium species are incorporated within the C–S–H gel (Hemstad *et al.*, 2024 ▸).

**Table 7:** Direct RQPA results for PC-20Qz paste (wt%) at different times from *in situ* Mo *K*α_1_ XRPD

Phase	*t*_0_	3 h	1 d	3 d	7 d	28 d
C_3_S	51.2	47.8 (3)	17.5 (6)	8.2 (2)	7.6 (9)	4.9 (6)
C_2_S	7.6	8.2 (4)	9.7 (4)	11.5 (3)	10.5 (6)	8.0 (7)
C_3_A	3.8	3.8 (2)	2.7 (3)	0.6 (2)		
C_4_AF	9.2	7.6 (4)	7.6 (4)	6.2 (5)	4.8 (5)	3.8 (4)
Cc	4.8	4.6	5.7	7.5	6.2	6.5
Qz	20.2	21.7 (2)	27.0 (2)	28.6 (3)	29.4 (3)	31.0 (3)
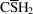	3.2	3.0 (2)				
AFt		2.6 (2)	12.1	12.3 (1)	12.5 (4)	13.0 (5)
CH		0.8 (1)	17.8 (2)	22.6 (2)	24.2 (3)	26.0 (3)
Hc				2.3 (1)	2.7 (1)	2.0 (1)
Mc				1.4 (2)	2.2 (2)	4.7 (3)

**Table 8:** RQPA for the PC-20Qz paste (wt%) at different times from *in situ* Mo *K*α_1_ LXRPD (values referred to 100 g of paste) The calculated amounts of amorphous phases are given in italics. The calculated amounts of crystalline hydrated phases are also given for comparison purposes.

Phases	*t*_0_	3 h	1 d	3 d	7 d	28 d
C_3_S	36.6	33.7	10.0	4.4	4.0	2.4
C_2_S	5.4	5.8	5.5	6.1	5.5	3.9
C_3_A	2.7	2.6	1.6	0.3		
C_4_AF	6.6	5.4	4.3	3.3	2.5	1.9
*Cc*	3.4	3.2	3.3	4.0	3.3	3.2
Qz	14.4	15.3	15.4	15.2	15.4	15.1
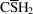	2.3	2.1				
*H_2_O*	*28.6*	*27.0*	*14.3*	*11.7*	*10.6*	*8.8*
AFt/AFt_calc_†		1.8/3.8	6.9/11.2	6.5/11.2	6.5/11.2	6.3/11.2
CH/CH_calc_		0.6/1.0	10.1/10.2	12.0/12.1	12.7/12.2	12.7/12.9
Hc/Hc_calc_				1.2/2.7	1.4/3.3	1.0/3.3
Mc/Mc_calc_				0.8/1.2	1.2/2.1	2.3/2.8
*C–S–H_calc_*		*1.5*	*26.6*	*31.5*	*33.2*	*38.1*
*Fe–Si–H_calc_*		*1.1*	*2.1*	*3.1*	*3.8*	*4.4*
Factor	0.714	0.704	0.570	0.532	0.524	0.486

† See footnote to Table 6.

**Table 9:** Direct Rietveld quantitative phase analysis results for PC-20LS paste (wt%) at different times from *in situ* Mo *K*α_1_ XRPD

Phase	*t*_0_	3 h	1 d	3 d	7 d	29 d
C_3_S	51.2	46.7 (3)	16.5 (3)	7.8 (4)	6.1 (4)	4.6 (4)
C_2_S	7.6	8.5 (3)	11.4 (4)	11.8 (7)	11.6 (4)	11.0 (5)
C_3_A	3.8	3.8 (3)	2.7 (2)	0.6 (2)		
C_4_AF	9.2	8.2 (4)	8.3 (4)	6.6 (4)	6.1 (4)	5.5 (5)
Cc	24.6	26.4	32.3	34.9	35.1	36.1
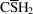	3.2	3.0 (1)				
Others	0.4					
AFt		2.7 (2)	11.5 (3)	12.2 (3)	12.8 (3)	12.0 (3)
CH		0.7 (1)	17.4 (2)	22.3 (2)	23.6 (2)	25.0 (3)
Hc				2.2 (1)	2.4 (1)	1.7 (1)
Mc				1.5 (2)	2.3 (2)	4.1 (2)

**Table 10:** RQPA for the PC-20LS paste (wt%) at different times from *in situ* Mo *K*α_1_ LXRPD (values referred to 100 g of paste) The calculated amounts of amorphous phases are given in italics. The calculated amounts of crystalline hydrated phases are also given for comparison purposes.

Phase	*t*_0_	3 h	1 d	3 d	7 d	29 d
C_3_S	36.6	33.1	9.5	4.2	3.3	2.4
C_2_S	5.4	6.0	6.6	6.4	6.2	5.7
C_3_A	2.7	2.7	1.5	0.3		
C_4_AF	6.6	5.8	4.8	3.6	3.3	2.9
Cc	17.4	18.7	18.5	18.8	18.7	18.8
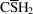	2.3	2.2				
Others	0.4					
*H_2_O*	*28.6*	*27.0*	*14.7*	*11.6*	*10.8*	*10.3*
AFt/AFt_calc_†		1.9/2.3	6.6/10.4	6.6/10.4	6.8/10.4	6.2/10.4
CH/CH_calc_		0.5/1.3	10.0/10.4	12.0/12.2	12.5/12.5	13.0/12.8
Hc/Hc_calc_				1.2/2.5	1.3/3.1	0.9/3.1
Mc/Mc_calc_				0.8/1.2	1.2/1.8	2.1/2.2
*C–S–H_calc_*		*1.4*	*26.2*	*31.6*	*32.9*	*34.2*
*Fe–Si–H_calc_*		*0.7*	*1.7*	*2.8*	*3.1*	*3.5*
Factor	0.714	0.709	0.574	0.540	0.532	0.521

† See footnote to Table 6.

**Table 11:** Comparison of µCT and RQPA results in vol.% for PC-525 The values for the µCT study have been renormalized to exclude the porosities. Data for PC-425 (Shirani *et al.*, 2024 ▸) are also given for comparison.

		µCT		LXRPD	
Hydration time	Components	PC-525	PC-425	PC-525	PC-425
*t*_0_	H_2_O			63.3	64.4
	UCP			36.7	35.6
					
12 h	HP/H_2_O	80.0	77.2		
	UCP	20.0	22.8		
					
1 d	HP/H_2_O	82.9	79.9	81.6	78.5
	UCP	17.1	20.1	18.4	21.5
					
3 d	HP/H_2_O	85.1	84.0	86.2	84.1
	UCP	14.9	16.0	13.8	15.9
					
7 d	HP/H_2_O	86.1	84.8	88.1	85.5
	UCP	13.9	15.2	11.9	14.5
					
∼28 d	HP/H_2_O	87.2		90.7	
	UCP	12.8		9.3	
					
Later age	HP/H_2_O	88.6	86.6	91.1	87.6
	UCP	11.4	13.4	8.9	12.4

**Table 12:** Degree of hydration (%) for PC-525, PC-20Qz and PC-20LS pastes from the RQPA normalized results

Phase	Paste	3 h	1 d	3 d	7 d	∼28 d†
C_3_S	PC-525	5	70	82	83	88^*a*^
C_3_S	PC-20Qz	8	72	88	89	93^*b*^
C_3_S	PC-20LS	10	74	88	91	93^*c*^
						
C_2_S	PC-525					∼20^*a*^
C_2_S	PC-20Qz					∼25^*b*^
C_2_S	PC-20LS					∼0
						
C_3_A	PC-525		43	78	100	100^*a*^
C_3_A	PC-20Qz	4	46	89	100	100^*b*^
C_3_A	PC-20LS	1	44	88	100	100^*c*^
						
C_4_AF	PC-525	12	22	42	48	61^*a*^
C_4_AF	PC-20Qz	18	34	50	62	72^*b*^
C_4_AF	PC-20LS	11	28	46	50	57^*c*^

† No. of days of hydration: superscript *a* indicates 31 d, superscript *b* 28 d and superscript *c* 29 d.

## Data Availability

All raw data used in this paper (calorimetry, LXRPD and X-ray µCT scan files) are openly accessible on Zenodo at https://doi.org/10.5281/zenodo.10390818.
